# mRNA 3′ UTRs direct microRNA degradation to participate in imprinted gene networks and regulate growth

**DOI:** 10.1101/gad.353479.125

**Published:** 2026-04-01

**Authors:** Daniel H. Lin, Lara E. Elcavage, Ekaterina Khalizeva, David P. Bartel

**Affiliations:** 1 Whitehead Institute for Biomedical Research, Cambridge, Massachusetts 02142, USA;; 2 Howard Hughes Medical Institute, Cambridge, Massachusetts 02142, USA;; 3 Department of Biology, Massachusetts Institute of Technology, Cambridge, Massachusetts 02139, USA;; 4 Harvard-Massachusetts Institute of Technology MD-PhD Program, Harvard Medical School, Boston, Massachusetts 02115, USA

**Keywords:** Zswim8, gene regulation, imprinting; miRNA, target-directed miRNA degradation

## Abstract

MicroRNAs direct downregulation of target mRNAs. Sometimes, however, this regulatory paradigm inverts, and a target RNA triggers degradation of a microRNA. This target-directed microRNA degradation (TDMD) requires ZSWIM8. *Zswim8*^−/−^ mice exhibit reduced growth and perinatal lethality, accompanied by stabilization of >40 microRNAs. Nonetheless, studies of TDMD function in mammals have been limited because only two TDMD-triggering RNAs have been identified in mice. Here, we computationally identify and validate five new TDMD-triggering sites in mouse models. One site in *Atp6v1g1* and two sites in *Lpar4* direct degradation of miR-335-3p, showing that in mammals, two sites in the same transcript and multiple sites in different transcripts can collaborate to destabilize a microRNA. Moreover, sites in *Plagl1* and *Lrrc58* direct degradation of miR-322 and miR-503, respectively. Mice lacking the *Plagl1* and *Lrrc58* sites were smaller, demonstrating that target-directed degradation of miR-322 and miR-503 promotes growth. Both miR-335-3p and *Plagl1* are maternally imprinted, implying their participation in parental conflict, but their corresponding triggers or target microRNA partners are not imprinted. Thus, 3′ UTRs can participate in parental conflict not only by regulating protein production but also by engaging TDMD to access an additional layer of regulation within a network of imprinted and biallelic genes.

## Abstract (precis)

In this study, Lin et al. identify trigger RNAs that drive target-directed microRNA degradation (TDMD) of ZSWIM8-sensitive microRNA. They describe instances in which multiple trigger RNAs or TDMD sites can collaboratively destabilize a miRNA, as well as highlight a link between the TDMD pathway and genomic imprinting.

MicroRNAs (miRNAs) are ∼22 nt RNAs that specify posttranscriptional gene repression (Bartel 2018). Mammalian genomes encode >500 miRNA genes (Clarke et al. 2025), and each miRNA can target hundreds of different mRNAs across the transcriptome, such that in aggregate, most human mRNAs are conserved targets of at least one miRNA (Friedman et al. 2009). Reinforcing this broad role for miRNAs, mice that lack either individual miRNAs or several members of the same miRNA family possess a wide spectrum of phenotypes (Bartel 2018). Precise control of miRNA expression is also critical across development, as overexpression of miRNAs can also result in developmental defects or disease (Kloosterman and Plasterk 2006; Sayed and Abdellatif 2011; Concepcion et al. 2012).

Each miRNA acts in concert with an Argonaute (AGO) protein, which envelops the miRNA and shapes its interactions with target mRNAs (Elkayam et al. 2012; Schirle and MacRae 2012; Faehnle et al. 2013; Schirle et al. 2014). The miRNA 5′ and 3′ ends bind specific pockets in AGO, which largely shields these ends from cellular exonucleases. As a result of this protection, miRNAs are typically very stable, with median half-lives of 34 h in mouse embryonic fibroblasts (MEFs), compared with median half-lives of 2 h for mRNAs (Kingston and Bartel 2019; Eisen et al. 2020). Within the Ago–miRNA complex, the miRNA recognizes mRNA targets primarily through base-pairing between nucleotides 2–7 of the miRNA (called the miRNA seed) and sites within the 3′ UTRs of the mRNA. This seed-pairing can be augmented on either end by additional pairing to miRNA nucleotide 8 or the presence of a target A nucleotide across from miRNA nucleotide 1 (Lewis et al. 2005; Grimson et al. 2007; Schirle et al. 2014, 2015). Although not typically observed, additional pairing between the target and the 3′ region of the miRNA can supplement seed-pairing to increase affinity for the target (supplementary pairing) or compensate for defects in base-pairing in the seed region (compensatory pairing) (Brennecke et al. 2005; Grimson et al. 2007; Bartel 2009). Stable association of the Ago–miRNA complex with the target RNA typically leads to recruitment of TNRC6, which in turn recruits deadenylation complexes that shorten the mRNA poly(A) tail. Pairing to the central nucleotides 9 and 10 is usually sterically occluded by AGO, but in conjunction with a conformational change, complete complementarity between the miRNA and the target activates AGO2 endonuclease activity, which slices the target RNA (Hutvágner and Zamore 2002; Sheu-Gruttadauria et al. 2019; Mohamed et al. 2025).

In rare cases, an inversion of this regulatory paradigm can occur, wherein the target RNA triggers selective decay of the miRNA. This target-directed miRNA degradation (TDMD) is mediated by a Cullin–Ring E3 ubiquitin ligase complex consisting of the substrate receptor ZSWIM8, adapter proteins ELOB and ELOC, and CUL3, which recruits additional ubiquitylation factors (Han et al. 2020; Shi et al. 2020). Polyubiquitylation of AGO causes its degradation by the proteasome, leaving the miRNA susceptible to cellular nucleases while freeing the TDMD-triggering RNA (or TDMD trigger) to direct additional cycles of TDMD (Han et al. 2020; Shi et al. 2020).

TDMD was first observed in response to either synthetic RNA targets with extensive complementarity (Ameres et al. 2010) or viral transcripts that direct the degradation of cellular miRNAs that would otherwise slow viral replication (Cazalla et al. 2010; Libri et al. 2012; Marcinowski et al. 2012; Lee et al. 2013). More recently, TDMD has been described for a handful of endogenously encoded sites (e.g., two in mice, seven in *Drosophila*, and one in worms) (Bitetti et al. 2018; Kleaveland et al. 2018; Kingston et al. 2022; Sheng et al. 2023; Hiers et al. 2025; Grimme et al. 2026), but molecular analyses of the effects of ZSWIM8 disruption in diverse contexts suggest the existence of many more that have yet to be found (Han et al. 2020; Shi et al. 2020, 2023; Kingston et al. 2022; Jones et al. 2023; Stubna et al. 2025).

In mice, loss of ZSWIM8 results in heart and lung development defects, reduced growth, and perinatal lethality, suggesting a critical role for TDMD in development (Jones et al. 2023; Shi et al. 2023). Accompanying these phenotypes, >40 miRNAs increase in abundance in various tissues of the mouse embryo, suggesting that they may be substrates of endogenous TDMD (Jones et al. 2023; Shi et al. 2023). However, only two murine transcripts have been identified and validated as endogenous transcripts that specify TDMD in vivo: The *Nrep* mRNA directs miR-29 degradation, and the *Cyrano* long noncoding RNA (lncRNA) directs miR-7 degradation (Bitetti et al. 2018; Kleaveland et al. 2018). *Serpine1* and *BCL2L11* mRNAs have also been reported as TDMD triggers for miR-30c/30e and miR-221/222, respectively, but these miRNAs are not significantly upregulated in *Zswim8*^−/−^ embryos (Ghini et al. 2018; Li et al. 2021; Jones et al. 2023; Shi et al. 2023). Determining whether the remaining ZSWIM8-sensitive miRNAs are also TDMD substrates requires identification of transcripts that direct their degradation.

The most notable feature of the *Cyrano* and *Nrep* sites that trigger TDMD is their extensive complementarity to the 3′ region of their respective miRNAs (Bitetti et al. 2018; Kleaveland et al. 2018). Indeed, these sites are the most extensively paired sites in the transcriptome for either miRNA (Supplemental Fig. S1A,B). The prevailing paradigm is that this extensive, TDMD-triggering 3′ complementarity is distinct from 3′ supplementary pairing and induces a conformational change in the Ago–miRNA–target ternary complex that can be specifically recognized by ZSWIM8, but the molecular details of such recognition are poorly understood (Sheu-Gruttadauria et al. 2019; Buhagiar and Kleaveland 2024). In support of this paradigm, mutations that attenuate either seed-pairing or extensive complementarity disrupt TDMD (Kleaveland et al. 2018; Sheu-Gruttadauria et al. 2019).

Although most genes are expressed equally from both sets of chromosomes, a few are preferentially expressed from one allele, which results in an asymmetric contribution from the paternal and maternal alleles (McGrath and Solter 1984; Surani et al. 1984; Cattanach and Kirk 1985; Cleaton et al. 2014; Peters 2014; Tucci et al. 2019). These imprinted genes gain robust epigenetic modifications in either the maternal or paternal germline that result in their silencing in the offspring. In mice, >200 such imprinted genes have been reported (Tucci et al. 2019). Among vertebrates, the process of imprinting is restricted to therian mammals, suggesting a link between imprinting and resource allocation in utero (Cleaton et al. 2014; Tucci et al. 2019). Kinship theory posits that the interests of the two parental genomes within offspring can conflict with respect to the use of maternal resources to support the growth of the fetus or newborn (Haig and Westoby 1989; Moore and Haig 1991; Tucci et al. 2019). For example, a murine gene that causes more maternal resources to be used to promote growth of the fetus or pup at the expense of either littermates or future litters will favor the interests of the paternal genome, whereas a gene that saves maternal resources for the benefit of littermates or future litters will favor the interests of the maternal genome. In this scenario, in which the fitness of an allele can differ depending on its inheritance pattern, imprinting provides the means by which the parents can favor the interests of their chromosomes (Peters 2014; Tucci et al. 2019). In support of this hypothesis, many maternally imprinted, paternally expressed genes increase the allocation of maternal resources to promote growth of the fetus or newborn, whereas paternally imprinted, maternally expressed genes limit growth (Peters 2014).

Interestingly, miRNAs that emerged around the time of the last common ancestor of placental mammals are highly enriched for imprinted loci, implying that miRNAs were enlisted early to do battle in parental genome conflicts. Indeed, of the 88 miRNA families conserved among placental mammals but absent in fish, >40% are imprinted. Of these, at least 40% are ZSWIM8-sensitive (Jones et al. 2023; Shi et al. 2023), suggesting that TDMD may also have been co-opted into this genomic conflict.

Here, we identified four additional endogenous triggers for three ZSWIM8-sensitive miRNAs, thereby tripling the number of known triggers in mammals. Of these four triggers, one is maternally imprinted and two direct the destruction of a maternally imprinted miRNA, thereby strengthening the link between the TDMD pathway and genomic imprinting.

## Results

### Computational identification of triggers for ZSWIM8-sensitive miRNAs

Although each miRNA typically possesses hundreds to thousands of potential target sites throughout the transcriptome, few of these sites, if any, trigger TDMD, creating a fundamental challenge as to how to identify the exceedingly rare sites that are effective TDMD triggers. The first endogenous TDMD triggers were identified through their canonical seed-pairing combined with their unusually extensive pairing to the miRNA 3′ region. Subsequent studies have focused on sites that resemble these few known and characterized examples. These sites have a few unifying features: (1) canonical seed-pairing, (2) extensive complementarity to the miRNA 3′ region, (3) high gene expression, and (4) evolutionary conservation.

Searching for sites with these characteristics has been most productive in *Drosophila*, identifying triggers for about half of the Dora-sensitive miRNAs in *Drosophila* S2 cells (Kingston et al. 2022). Accordingly, we used a similar scheme to search for candidate TDMD sites within the transcriptome of MEFs. For one branch of this search, we considered only sites with conserved miRNA seed matches in mRNA 3′ UTRs (Supplemental Fig. S1A). In another branch, we considered all sites with canonical seed matches in mRNA 3′ UTRs and annotated lncRNAs (Supplemental Fig. S1B). Sites were filtered for expression in MEFs, and extensively complementary targets were prioritized with a scoring scheme that (1) awarded points for Watson–Crick–Franklin base-pairing to the 3′ region of the miRNA, (2) penalized noncontiguous base-pairing, (3) awarded additional points for pairing to nucleotides near the end of the miRNA, and (4) penalized large bulges or internal loops in predicted pairing between the miRNA and target. As an orthogonal metric for pairing quality, we also predicted the folding energy for pairing between the miRNA-binding site and the miRNA 3′ region, as was done in *Drosophila* (Kingston et al. 2022). Both approaches confirmed that the previously characterized miRNA-binding sites in *Cyrano* and *Nrep* possessed exceptional pairing to the 3′ region of miR-7 and miR-29b, respectively (Supplemental Fig. S1A,B). Top-scoring candidates for other miRNAs were tested by knocking down or knocking out the site predicted to direct miRNA degradation and monitoring the effect on the miRNA. This approach identified triggers for miR-335-3p, miR-322, and miR-503.

### Multiple TDMD sites, from the same and different trigger transcripts, direct degradation of miR-335-3p

**Figure 1.**
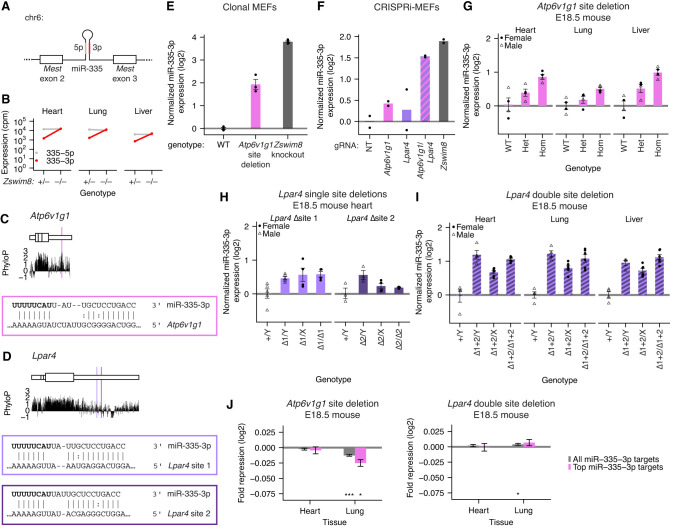
Sites in *Atp6v1g1* and *Lpar4* 3′ UTRs collaborate to mediate TDMD in MEFs and in vivo. (*A*) Structure of the *Mest* gene, which harbors the *Mir335* gene within intron 2. (*B*) Expression of miR-335-3p and miR-335-5p strands in embryonic day 18.5 (E18.5) hearts, lungs, and livers of *Zswim8*^−/+^ and *Zswim8*^−/−^ mice (Shi et al. 2023). (cpm) Counts per million miRNA reads. (*C*) mRNA context, evolutionary conservation, and base-pairing diagram for the miR-335-3p trigger site in *Atp6v1g1.* (*Top*) The *Atp6v1g1* mRNA. (Larger rectangles) Coding sequence, (smaller rectangles) UTRs. Vertical lines indicate exon boundaries. (*Middle*) PhyloP scores from a mammalian 60 way alignment (Pollard et al. 2010) plotted in 5 nt bins. (*Bottom*) Pairing diagram depicting complementarity between miR-335-3p and its trigger site in the *Atp6v1g1* 3′ UTR. Vertical lines indicate W–C–F pairing, and colon indicates G:U wobble-pairing. (*D*) mRNA context, evolutionary conservation, and base-pairing diagrams for the miR-335-3p trigger sites in *Lpar4*; otherwise, as in *C*. (*E*) Function of the miR-335-3p trigger site in *Atp6v1g1*. Plotted is the quantification of miR-335-3p (as measured by small RNA sequencing [sRNA-seq]) from clonal MEF cell lines with either homozygous deletion of the miR-335-3p trigger site or *Zswim8* knockout. To account for clone-to-clone variability in miR-335 production, the depth-normalized expression of miR-335-3p was normalized to the expression of its cotranscribed miR-335-5p strand. Each point represents the fold change of normalized expression relative to the mean of normalized expression in WT samples. *n* = 3–4 clonal lines per genotype. Error bars indicate standard error. (*F*) Function of the *Atp6v1g1* and *Lpar4* trigger transcripts. Plotted is the quantification of miR-335-3p expression, as measured by sRNA-seq following CRISPRi knockdown of either *Atp6v1g1*, *Lpar4*, both mRNAs, or *Zswim8* (*n* = 2 biological replicates); otherwise, as in *E*. (*G*) In vivo TDMD activity of the miR-335-3p trigger site in *Atp6v1g1.* Shown is the quantification by Northern blot of miR-335-3p expression (normalized to miR-335-5p expression) in E18.5 hearts, lungs, and livers of mice harboring deletions of the miR-335-3p TDMD site in *Atp6v1g1* (*Atp6v1g1*^−50^). (Circles) Female animals, (triangles) male animals. *n* = 4 replicates per tissue for each genotype. (*H*) In vivo TDMD activity of site 1 or site 2 in *Lpar4* ([site 1 mutant] *Lpar4*^−173^, [site 2 mutant] *Lpar4*^−69+36^); otherwise, as in *G*. *n* = 3–5 replicates per tissue for each genotype and sex. (*I*) In vivo activity of both miR-335-3p trigger sites in *Lpar4* ([site 1 and 2 mutant] *Lpar4*^−197^); otherwise, as in *G*. *n* = 4 replicates per tissue for male samples; *n* = 5 replicates per tissue for female samples. (*J*) The influence of trigger sites within *Atp6v1g1* and *Lpar4* on the levels of predicted miR-335-3p targets in vivo. Two sets of targets were analyzed: “all predicted targets” and “top predicted targets” (top 10% of predicted targets, as determined by TargetScan) (Agarwal et al. 2015). For each set of predicted targets, a set of nontarget transcripts, matched for 3′ UTR length, was sampled at a 1:1 ratio, and the distributions of log_2_ fold changes of mutant mice compared with WT littermates were compared between predicted target and nontarget cohorts. The repression metric plotted is the difference between the median log_2_ fold change (mutant/WT) of the target cohort and that of the nontarget cohort. This analysis was repeated 20 additional times, sampling new nontarget cohorts with each iteration. Plotted is the mean repression metric and median *P*-value across the 21 iterations. Error bars indicate standard deviation. For all figures, (*) *P* < 0.05, (**) *P* < 0.005, (***) *P* < 0.0005, Mann–Whitney *U*-test comparing the distribution for predicted targets and that of their nontarget cohort.

Mouse miR-335-3p is one of the most potently and broadly upregulated miRNAs in *Zswim8*^−/−^ embryonic tissues (Jones et al. 2023; Shi et al. 2023). The *Mir335* pri-miRNA hairpin is located within an intron of the maternally imprinted gene *Mest* and is therefore only expressed from the paternal allele (Fig. 1A; Hiramuki et al. 2015). Typically, a miRNA is generated from an RNA hairpin that is processed in successive cleavage steps to yield a duplex of two ∼22 nt strands derived from the 5′ and 3′ arms of the hairpin (Bartel 2018; Kim et al. 2025). One strand of each duplex associates with an AGO protein and serves as the functional guide RNA, whereas the other strand, known as the passenger strand, is discarded and rapidly degraded. Most miRNA duplexes exhibit a bias as to which strand associates with AGO and which strand is discarded, and the relative accumulation of the guide and passenger strands reflects this bias (Bartel 2018). For miR-335, TDMD also helps to determine the more prominent strand (Jones et al. 2023; Shi et al. 2023). For this miRNA, the strand from the 3′ arm of the hairpin, designated miR-335-3p, typically accumulates at much lower levels than its coproduced strand from the 5′ arm of the hairpin, miR-335-5p, but this ratio is a consequence of the potent, strand-specific turnover of miR-335-3p by TDMD (∼7.4-fold in MEFs; up to ∼17.8-fold in the embryonic day 18.5 [E18.5] stomach) (Fig. 1A,B; Shi et al. 2023). Thus, miR-335-3p is the more abundant strand in *Zswim8* knockout tissues—a rare example in which TDMD causes apparent “arm switching.”

We identified a site in the 3′ UTR of *Atp6v1g1* and two sites in the 3′ UTR of *Lpar4* as the top-scoring candidate TDMD sites for miR-335-3p (Fig. 1C,D; Supplemental Fig. S1A,B). The potential role of the *Atp6v1g1* site in MEFs was tested by Cas9-mediated deletion of the site using two flanking guide RNAs, which yielded ∼50 nt deletions encompassing the miR-335-3p site. Although substantial clone-to-clone variability in expression was observed for both strands of miR-335, presumably caused by differences in production of the miRNA hairpin, the posttranscriptional effects of *Atp6v1g1* site deletion on miR-335-3p could be isolated by normalizing to the expression of the coproduced miR-335-5p strand. After this normalization, the effect of *Atp6v1g1* site deletion on miR-335-3p expression was only ∼25% of the effect of *Zswim8* knockout, suggesting that another trigger was acting partially redundantly in MEFs (Fig. 1E; Supplemental Fig. S2A). Suspecting that the sites in *Lpar4* might be responsible for the remaining effect, a similar deletion strategy was attempted for these two sites, but we were unable to obtain clones in which the *Lpar4* sites were deleted on all alleles. Therefore, we turned to CRISPRi knockdown experiments. In these experiments, we observed weak effects when either *Atp6v1g1* or *Lpar4* was individually targeted but nearly the full effect of the *Zswim8* knockdown when both genes were targeted (Fig. 1F; Supplemental Fig. S2B). We conclude that these two transcripts collaborate to direct the degradation of miR-335-3p in MEFs.

Although *Atp6v1g1* and *Lpar4* contribute similarly to miR-335-3p turnover, they are expressed at substantially different levels. In MEFs, *Atp6v1g1* expression is ∼42-fold greater than *Lpar4* expression, as measured by RNA-seq (179 vs. 4.2 transcripts per million [TPM]) (Supplemental Fig. S2C). Even after accounting for the presence of two sites per *Lpar4* transcript, this difference in expression levels suggests more efficient turnover of miR-335-3p by *Lpar4* than by *Atp6v1g1*.

To more thoroughly validate the three TDMD sites in *Atp6v1g1* and *Lpar4*, we generated *Atp6v1g1* and *Lpar4* mutant mice with targeted deletions of the TDMD sites. The *Atp6v1g1*^−50^ mice possessed a 50 nt deletion encompassing the miR-335-3p site (Supplemental Fig. S3A). The allelic series of *Lpar4* mutant mice possessed deletions that disrupted either an individual site (*Lpar4*^−173^ [site 1 mutant] or *Lpar4*^−69+36^ [site 2 mutant]) or both sites (*Lpar4*^−197^) (Supplemental Fig. S3B).

We bred mice heterozygous for the *Atp6v1g1*^−50^ allele and examined miR-335-3p levels in their progeny at E18.5. Homozygous deletion of the TDMD site in *Atp6v1g1* caused an increase in miR-335-3p levels in all three embryonic tissues examined—heart, lung, and liver (Fig. 1G; Supplemental Fig. S3C). Heterozygous deletion of the site caused an intermediate level of miR-335-3p elevation, indicating a dosage-dependent effect of TDMD activity by *Atp6v1g1*. The increases in miR-335-3p observed in homozygous *Atp6v1g1*^−50^ tissues represented 8%, 6%, and 18% of that reported in *Zswim8*^−/−^ tissues compared with *Zswim8*^−/+^ tissues (heart: 1.8-fold vs. 10.5-fold; lung: 1.4-fold vs. 7.8-fold; liver: 2.0-fold vs. 6.6-fold) (Shi et al. 2023), which suggested that at least one additional trigger (perhaps *Lpar4*) might be required to achieve the full effect of ZSWIM8.

To assess the contribution of TDMD sites in *Lpar4* to miR-335-3p degradation, we bred hemizygous males with heterozygous females for each of the alleles in the allelic series. Deletion of either individual TDMD site in *Lpar4* caused 1.4-fold and 1.3-fold increases in miR-335-3p levels in male E18.5 hearts for site 1 and site 2, respectively (Fig. 1H; Supplemental Fig. S3D,E). Deletion of both TDMD sites in *Lpar4* caused a greater increase in miR-335-3p levels (2.2-fold in the heart, 2.2-fold in the lung, and 2.1-fold in the liver), accounting for 13%–20% of the reported effect in *Zswim8*^−/−^ tissues (Fig. 1I; Supplemental Fig. S3F; Shi et al. 2023).

Deletion of the TDMD trigger sites in either *Atp6v1g1* or *Lpar4* did not significantly alter the accumulation of either mRNA, as quantified by RNA-seq (Supplemental Fig. S4A). These observations were consistent with results observed upon disruption of the miR-7 trigger site in Cyrano or disruption of other TDMD trigger sites in *Drosophila* S2 cells (Kleaveland et al. 2018; Kingston et al. 2022) and add to the body of evidence that trigger sites typically do not mediate substantial mRNA decay while engaged in the TDMD pathway.

miR-335-3p targets were mildly downregulated in *Atp6v1g1*^−50^ lungs, as expected due to the upregulation of miR-335-3p (Fig. 1J; Supplemental Fig. S4B,C). However, there was no significant downregulation of miR-335-3p targets in *Lpar4*^−197^ lungs despite a slightly greater upregulation of miR-335-3p, possibly due to differences in the expression pattern of the two transcripts throughout the tissue (Fig. 1J; Supplemental Fig. S4D). Similarly, there was no significant downregulation of miR-335-3p targets in *Atp6v1g1*^−50^ hearts or *Lpar4*^−197^ hearts (Fig. 1J; Supplemental Fig. S4C,D). Presumably, a greater signal for regulation of miR-335-3p targets would be detectable upon loss of both TDMD triggers.

These observations in *Atp6v1g1* and *Lpar4* mutant mice confirmed the independent function of these two triggers for miR-335-3p TDMD in vivo. Thus, in mice, a miRNA can be targeted by multiple partially redundant TDMD sites—both in a single transcript and across multiple transcripts. Consistent with observations in cell culture, these two transcripts contributed similarly to the degradation of miR-335-3p in animals despite vast differences in their expression (Supplemental Fig. S2C; Shi et al. 2023), suggesting the existence of unknown factors that might enhance the potency of some triggers.

### *Plagl1* and *Lrrc58* direct degradation of miR-322 and miR-503

**Figure 2.**
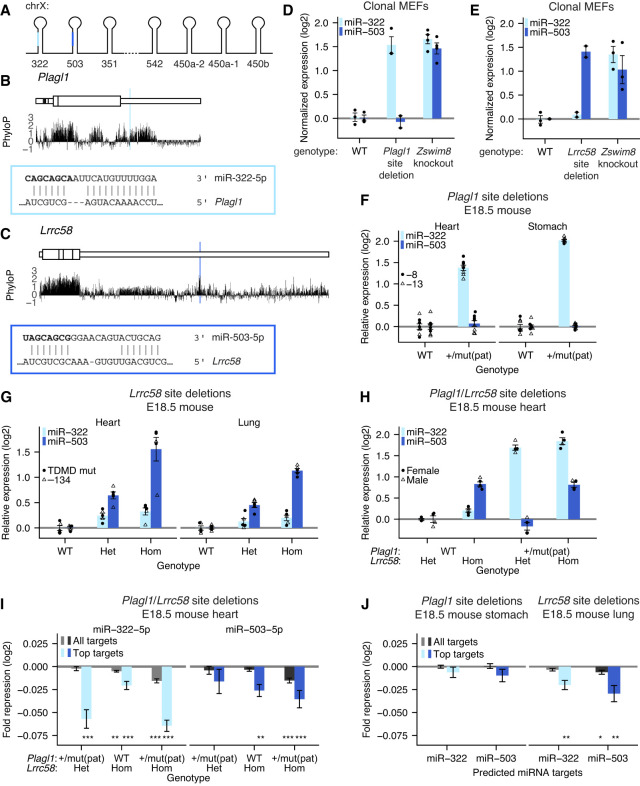
Sites in the 3′ UTRs of *Plagl1* and *Lrrc58* are the major TDMD triggers of miR-322-5p and miR-503-5p, respectively. (*A*) Organization of the X-linked cluster of seven miRNA genes, including genes for miR-322 and miR-503. The dashed line between the *Mir351* and *Mir542* hairpins denotes a longer genomic distance and loss of coordinated expression. (*B*) mRNA context, evolutionary conservation, and base-pairing diagram for the miR-322 trigger site within *Plagl1*; otherwise, as in Figure 1C. (*C*) mRNA context, evolutionary conservation, and base-pairing diagram for the miR-503 trigger site within *Lrrc58*; otherwise, as in Figure 1C. (*D*) Function of the miR-322 trigger site in *Plagl1*. To account for clone-to-clone variability in cluster expression, the depth-normalized expression of miR-322-5p was first normalized to the expression of cotranscribed miRNAs miR-322-3p, miR-503-3p, and miR-351-3p; otherwise, as in Figure 1E. *n* = 3–4 clonal lines per genotype. (*E*) Function of the miR-503 trigger site in *Lrrc58*; otherwise, as in *D*. *n* = 2–3 clonal lines per genotype. (*F*) In vivo activity of the miR-322 trigger site in *Plagl1*. Shown is the quantification by Northern blot of miR-322-5p or miR-503-5p expression in E18.5 heart and lung tissue of *Plagl1*-mutant mice harboring an 8 nt deletion or 22 nt deletion/7 nt insertion within the miR-322 TDMD site. *n* = 8 replicates per tissue for each genotype. (*G*) In vivo activity of the miR-503 trigger site in *Lrrc58*. Quantification of miR-322-5p or miR-503-5p expression by Northern blot in E18.5 heart and lung tissue of *Lrrc58* mutant mice harboring either a 134 nt deletion or a precise mutation of the miR-503 TDMD trigger site. *n* = 5 replicates per tissue for each genotype. (*H*) In vivo activity of both the miR-322 trigger site in *Plagl1* and the miR-503 trigger site in *Lrrc58*. Quantification of miR-322-5p or miR-503-5p expression by Northern blot in E18.5 heart tissue of mutant mice harboring deletions of the miR-322 and miR-503 TDMD trigger sites in *Plagl1* and *Lrrc58*. Genotypes are indicated *below* the *X*-axis. (*Lrrc58* het) Heterozygous mutant *Lrrc58*, (*Lrrc58* hom) homozygous mutant *Lrrc58*, (*Plagl1* WT) wild-type *Plagl1*, [*Plagl1* +/mut(pat)] heterozygous mutant *Plagl1* with paternal inheritance. *n* = 4–5 replicates for each genotype. (*I*) The influence of trigger sites within *Plagl1* and *Lrrc58* on the levels of predicted miR-322-5p or miR-503-5p targets in vivo; otherwise, as in Figure 1J. (*J*) The influence of trigger sites within *Plagl1* or *Lrrc58* on the levels of predicted miR-322-5p or miR-503-5p targets in vivo; otherwise, as in Figure 1J.

We also considered the potential TDMD triggers of miR-322 and miR-503, two miRNAs that have unusually short half-lives in mammalian cells (Rissland et al. 2011; Kingston and Bartel 2019). These two miRNAs are members of the extended miR-15/16 seed family and are coexpressed from a miRNA cluster located on the X chromosome (Fig. 2A). miR-322 has the same sequence in its seed region as other members of the miR-15/16 seed family, whereas miR-503 differs at position 8 of the seed region. As a result, these miRNAs are predicted to target partially overlapping but distinct cohorts of target mRNAs (Agarwal et al. 2015; McGeary et al. 2019). Despite these similarities in the seed region, the sequences of these miRNAs diverge substantially in their 3′ regions, implying that different trigger sites might direct their degradation. Indeed, a computational search for extensively paired conserved sites for miR-322 and miR-503 identified sites in the 3′ UTRs of different mRNAs—with a miR-322 site in the *Plagl1* 3′ UTR and a miR-503 site in the *Lrrc58* 3′ UTR found among the top hits (Fig. 2B,C; Supplemental Fig. S1A,B). Notably, the proposal that pairing to the site in *Lrrc58* directs degradation of miR-503 is fully consistent with results of scanning mutagenesis that identify the nucleotides of miR-503 responsible for its destabilization (Rissland et al. 2011).

Disruption of the site in *Plagl1* caused miR-322 to accumulate to levels comparable with *Zswim8* knockout, after accounting for clone-to-clone variability in cluster expression (Fig. 2D; Supplemental Fig. S5A). Likewise, disruption of the site in *Lrrc58* caused miR-503 to accumulate to levels comparable with *Zswim8* knockout, again after accounting for clone-to-clone variability in cluster expression (Fig. 2E; Supplemental Fig. S5B). Thus, in MEFs, these two mRNAs were the major TDMD triggers for their respective miRNAs.

We next assessed the extent to which *Plagl1* and *Lrrc58* triggered TDMD in the mouse embryo. We generated two *Plagl1* lines with either an 8 nt deletion (*Plagl1*^−8^) or a 22 nt deletion/7 nt insertion (*Plagl1*^−22+7^) that disrupted complementarity to the 3′ region of miR-322 (Supplemental Fig. S4C). Because *Plagl1* is maternally imprinted, heterozygous mice with a paternally inherited mutant allele (*Plagl1*^+/mut(pat)^) express only the mutant allele (Kamiya 2000; Piras et al. 2000). The increased miR-322 accumulation in *Plagl1*^+/mut(pat)^ embryonic tissues approached that observed when comparing *Zswim8*^−/−^ embryonic tissues with *Zswim8*^−/+^ embryonic tissues (heart: 2.6-fold vs. 2.7-fold; stomach: 4.0-fold vs. 5.7-fold) (Fig. 2F; Supplemental Fig. S5D,E; Shi et al. 2023).

Similarly, for *Lrrc58*, we generated three mouse lines containing either four substitutions in the miR-503 TDMD site (*Lrrc58*^TDMD mut^) or 134 or 402 nt deletions encompassing the site (*Lrrc58*^−134^ and *Lrrc58*^−402^, respectively) (Supplemental Fig. S5F). Disruption of the site via targeted substitutions or deletion of the entire site resulted in similar upregulation of miR-503 in E18.5 hearts and lungs, which resembled or surpassed that reported for the increased accumulation in *Zswim8*^−/−^ tissues compared with *Zswim8*^−/+^ tissues (heart: 3.5-fold vs. 2.5-fold; lung 3.6-fold vs. 1.8-fold) (Fig. 2G; Supplemental Fig. S5G,H; Shi et al. 2023). Similar to observations in *Atp6v1g1*^−50^ embryonic tissue, miR-503 was elevated by an intermediate amount in mice heterozygous for the *Lrrc58* site mutation, demonstrating dosage-dependent TDMD by *Lrrc58* (Fig. 2G).

The miR-322 fold change in *Plagl1* mutant tissues was somewhat less than that observed when comparing *Zswim8*^−/−^ with *Zswim8*^−/+^ tissues (Shi et al. 2020). Perhaps explaining this result, miR-322 is weakly but consistently upregulated in *Lrrc58* mutant samples (Fig. 2G), and miR-322 accumulation increased 3.6-fold in *Plagl1*^+/−22+7(pat)^, *Lrrc58*^−134/−134^ double-mutant heart samples (Fig. 2H; Supplemental Fig. S6A). Thus, whereas *Plagl1* and *Lrrc58* were the dominant TDMD triggers for miR-322 and miR-503, respectively, in these embryonic tissues, *Lrrc58* appeared to have weak TDMD activity for miR-322 as well.

Consistent with upregulation of miR-322 and miR-503 in the TDMD site mutants, predicted targets of miR-322 and miR-503 were more repressed in mutant E18.5 heart tissue (Fig. 2I; Supplemental Fig. S6B,C). For each miRNA, the greatest repression of predicted targets was observed in *Plagl1*/*Lrrc58* double mutants, which was consistent with the partially overlapping targetome of these two related miRNAs. We also observed repression of predicted miR-322 and miR-503 targets in *Lrrc58* mutant E18.5 lung tissue (Fig. 2J; Supplemental Fig. S7A,B). In contrast, we did not detect increased repression of miR-322 or miR-503 targets in *Plagl1* mutant E18.5 stomach tissue (Fig. 2J; Supplemental Fig. S7B), perhaps due to the lower expression of miR-322 in stomach tissue (Shi et al. 2023). Similar to the observations in *Atp6v1g1* and *Lpar4* mutants, we did not detect significant changes in *Plagl1* or *Lrrc58* mRNA levels in the *Plagl1* or *Lrrc58* mutant samples (Supplemental Figs. S6B, S7A,C,D).

### ZSWIM8 sensitivity is often, but not always, conserved among mammals

**Figure 3.**
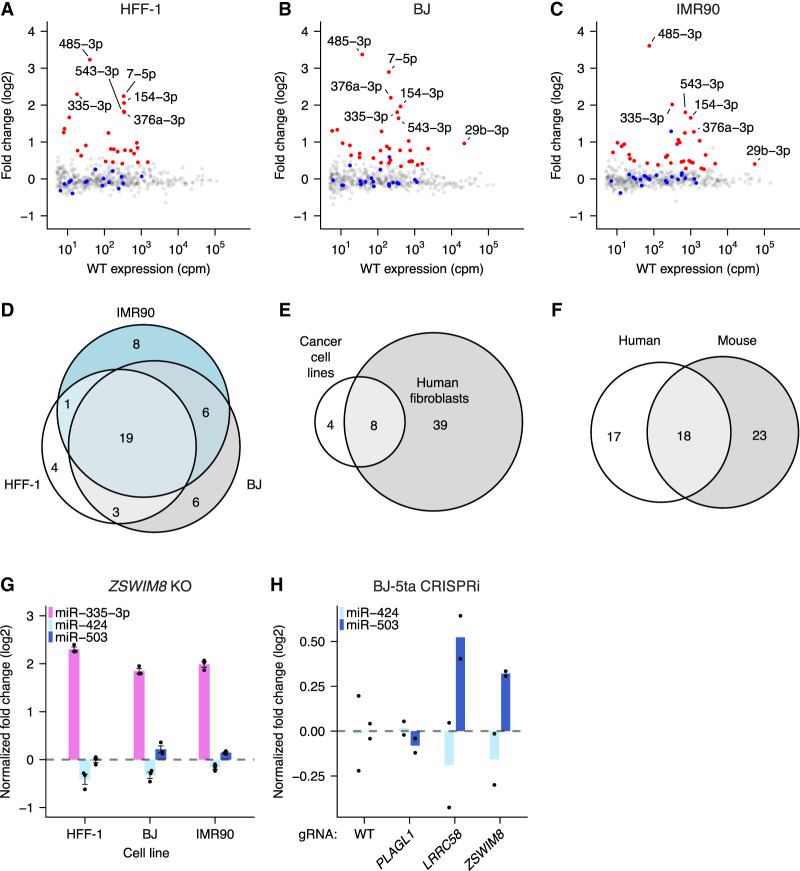
Evolutionary conservation of ZSWIM8 sensitivity. (*A*) ZSWIM8 sensitivity in HFF-1 cells. Plotted are fold changes of miRNA levels upon polyclonal knockout of *ZSWIM8*, as measured by sRNA-seq with analysis by DESeq2 (Love et al. 2014). *n* = 3 biological replicates. Values for which ZSWIM8 sensitivity was statistically significant are in red (Supplemental Table S1; Wang and Bartel 2023). (*B*) ZSWIM8 sensitivity in BJ cells; otherwise, as in *A*. (*C*) ZSWIM8 sensitivity in IMR90 cells; otherwise, as in *A*. (*D*) Overlap of ZSWIM8-sensitive miRNAs in three human fibroblast cell lines. (*E*) Overlap of miRNAs found to be ZSWIM8-sensitive in human fibroblasts with those found to be sensitive in human cancer cell lines (Shi et al. 2020). (*F*) Overlap of miRNAs determined to be ZSWIM8-sensitive in human fibroblast cell lines with those found to be sensitive in MEFs. (*G*) ZSWIM8 sensitivity of miR-335-3p, miR-424, and miR-503 in human fibroblast cell lines. Each point represents the fold change of normalized expression in ZSWIM8 knockdown cells, relative to the mean of normalized expression in WT samples after normalization to cluster members. *n* = 3 biological replicates per cell line. Bars represent the mean for each cell line. (*H*) Response of miR-503-5p and miR-424-5p to the knockdown of *PLAGL1*, *LRRC58*, or *ZSWIM8* in BJ-5ta cells; otherwise, as in *G*. *n* = 2 biological replicates per mRNA.

Although >50 miRNAs increase in expression in murine *Zswim8*^−/−^ embryonic tissues or cell lines, many fewer miRNAs have been observed to increase in expression in human cell lines; a total of 12 miRNAs were confidently annotated as increasing across five cell lines (Shi et al. 2020). The simplest explanation for this difference is that the human analyses have been performed in more restricted contexts—primarily in cancer cell lines. If so, perturbation of *ZSWIM8* in human fibroblast cell lines, which more closely resemble the previously assessed mouse fibroblast cell lines, might identify more TDMD substrates in human cells. Indeed, sequencing miRNAs from *ZSWIM8* knockout HFF-1, IMR90, and BJ human fibroblast lines identified 27, 34, and 34 upregulated miRNAs, respectively, after stringent statistical filtering (Fig. 3A–C; Supplemental Fig. S8A–C; Supplemental Table S1; Wang and Bartel 2023). Altogether, we identified 47 unique ZSWIM8-sensitive miRNAs, expanding the cohort of miRNAs reported to be ZSWIM8-sensitive in human cells by 39 (Fig. 3D,E). Of the 41 mouse ZSWIM8-sensitive miRNAs with clear human homologs, 18 miRNAs passed statistical thresholds in at least one human fibroblast line (Fig. 3F; Supplemental Table S2). Moreover, some that did not pass the statistical thresholds were nonetheless mildly upregulated (Supplemental Fig. S8D–F). Thus, we expect that this analysis provided a conservative lower bound on conservation of TDMD between the two species.

We next considered the evolutionary conservation of the newly identified TDMD sites. The *Lpar4* and *Atp6v1g1* genes are broadly conserved among vertebrates, but their miR-335 TDMD sites are present only in placental mammals (Fig. 1C,D; Supplemental Fig. S9A–C). This conservation is consistent with emergence of the *Mir335* gene after the divergence of placental mammals and marsupials. However, the three TDMD sites exhibit different patterns of conservation within placental mammals. The *Atp6v1g1* site is broadly conserved and is predicted to maintain its TDMD-triggering base-pairing pattern in nearly all placental species (Fig. 1C; Supplemental Fig. S9B). In a handful of species, such as guinea pigs, a single-nucleotide G-to-A substitution in *Atp6v1g1* across from miRNA nucleotide 15 is predicted to convert a G:U wobble into a canonical base pair and thereby strengthen the pairing to miR-335.

In contrast, the two sites in *Lpar4* have weaker evolutionary conservation (Fig. 1D; Supplemental Fig. S7C). In primates, the second site has frequently lost a canonical seed match, but this loss often appears to be compensated for by strengthened pairing to the first site, wherein G:U wobbles are replaced with canonical pairing. In contrast, moles lost substantial 3′ pairing in the first site while maintaining extensive complementarity in the second site. Thus, the presence of multiple sites targeting the same miRNA appears to have provided additional opportunity for compensation and for tuning the precise extent of TDMD over mammalian evolution.

Similar to miR-335, the miR-322/503 cluster arose soon after the divergence of placental mammals and marsupials and is conserved in nearly all placental animals. The miR-503 site within *Lrrc58* is also highly conserved throughout placental mammals and is the most conserved segment within the *Lrrc58* 3′ UTR (Fig. 2C). Moreover, the interaction between miR-503 and its TDMD trigger site is further supported by covariation, in which a single-nucleotide change at nucleotide 17 of monkey and ape miR-503 is accompanied by a change in the miR-503 binding site that preserves base-pairing (Supplemental Fig. S10A). Additionally, the miR-503 of New World monkeys possesses a change to A at nucleotide 16, which further extends complementarity between miR-503 and *Lrrc58*.

In contrast, the miR-322 trigger site appears to have a more varied evolutionary history. A seed match to miR-322 is present throughout mammalian *Plagl1* sequences, and some 3′ pairing is present in most mammalian species, suggesting that *Plagl1* is typically a miR-322 target and that the ancestral site possessed canonical seed as well as supplementary pairing (Supplemental Fig. S7C). However, the extensive complementarity observed in mice is present only among members of the *Muridae* family as well as within the *Afrotheria* clade. Within *Muridae*, two sequential changes appear to have led to the acquisition of extensive complementarity: (1) a change of miR-322 nucleotide 21 from the ancestral A to a G in a common ancestor of the *Muroidea* superfamily (including voles, hamsters, and jerboas), which resulted in complementarity to a C nucleotide in *Plagl1*, and (2) a change of the *Plagl1* sequence in a common ancestor of the *Muridae* family, which resulted in complementarity to miR-322 nucleotides 12–14. Thus, the ancestral miR-322 site may have been poised to acquire the ability to direct miR-322 degradation but appears to have acquired it only recently, in an ancestor of mice and rats, as well as in an ancestor of *Afrotheria*. Accordingly, in contrast to miR-335-3p and miR-503, we did not detect ZSWIM8 sensitivity of miR-424, the human ortholog of miR-322, suggesting that *Plagl1* TDMD activity does not extend to humans (Fig. 3G). Analogous results were obtained when knocking down newly identified TDMD triggers in BJ-5ta human fibroblasts (a telomerase immortalized derivative of the BJ cell line). Consistent with the predictions of our evolutionary analysis, we observed upregulation of miR-503 upon *LRRC58* knockdown but did not observe upregulation of miR-424 upon *PLAGL1* knockdown (Fig. 3H; Supplemental Fig. S8G–I). (Although ZSWIM8 sensitivity of miR-335-3p is conserved in humans [Fig. 3G], we were unable to assess the conservation of TDMD by human *ATP6V1G1* and *LPAR4* because miR-335-3p is not expressed in BJ-5ta fibroblasts.)

### *Plagl1-* and *Lrrc58*-directed degradation of miR-322 and miR-503 enhances growth of mice

**Figure 4.**
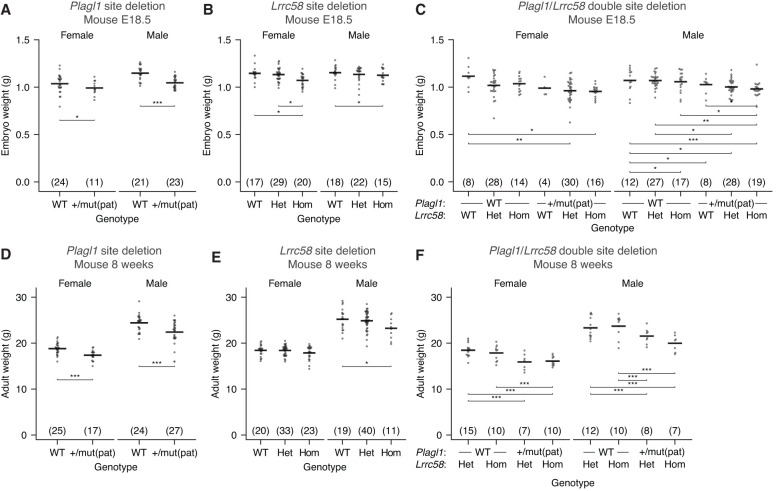
TDMD of miR-322 and miR-503 promotes growth. (*A*) Loss of *Plagl1*-directed miR-322 degradation reduces embryonic growth. Shown are the weights of E18.5 mouse embryos with either a wild-type (WT) or a mutant miR-322 trigger site in the expressed *Plagl1* allele. [mut(pat)] A paternally inherited *Plagl1* allele with a mutation in the miR-322 trigger site. The number of animals for each genotype is indicated in parentheses. Statistical testing was performed using a mixed linear-effects model. (*) *P* < 0.05, (**) *P* < 0.005, (***) *P* < 0.0005. (*B*) Loss of *Lrrc58*-directed miR-503 degradation reduces embryonic growth; otherwise, as in *A*. (WT) Wild type, (het) heterozygous mutant *Lrrc58*, (hom) homozygous mutant *Lrrc58*. (*C*) Loss of both *Plagl1*-directed miR-322 degradation and *Lrrc58*-directed miR-503 degradation further reduces embryonic growth. Plotted are the weights of E18.5 embryos generated from crossing *Lrrc58* het, *Plagl1* WT females with *Lrrc58* het, *Plagl1*^+/mut(pat)^ males; otherwise, as in *A* and *B*. (*D*) Loss of *Plagl1*-directed miR-322 degradation reduces the size of adult mice. Shown are the weights of 8 week old mice with either a wild-type (WT) or a mutant miR-322 trigger site at the expressed *Plagl1* allele [+/mut(pat)]; otherwise, as in *A*. (*E*) Loss of *Lrrc58*-directed miR-503 degradation reduces the size of adult mice. Plotted are the weights of 8 week old mice with either a wild-type, heterozygous mutant, or homozygous mutant miR-503 trigger site within *Lrrc58*; otherwise, as in *B*. (*F*) Loss of both *Plagl1*-directed miR-322 degradation and *Lrrc58*-directed miR-503 degradation further reduces the size of adult mice. Plotted are the weights of 8 week old mice generated from crossing *Lrrc58* hom, *Plagl1* WT females with *Lrrc58* het, *Plagl1*^+/mut(pat)^ males; otherwise, as in *C*.

*Zswim8*^−/−^ mice possess growth and developmental defects (Jones et al. 2023; Shi et al. 2023), but how deregulation of miRNA degradation contributes to each defect is unclear. Indeed, some or all of these defects might be attributed to disrupted degradation of non-AGO targets reported for this E3 ligase substrate receptor (Molina-Pelayo et al. 2022; Wang et al. 2023; Ren et al. 2024). To begin to link targeted degradation of specific miRNAs to their downstream consequences, we evaluated the effects of disrupting the TDMD trigger sites for miR-322 and miR-503. Mice with disrupted trigger sites within *Plagl1*, *Lrrc58*, or both triggers were viable and fertile but had reduced body size. At E18.5, *Plagl1* and *Lrrc58* mutant mice were 5.5% and 4.8% smaller by weight, respectively, compared with wild-type littermates (Fig. 4A,B). Furthermore, these effects were additive, as *Plagl1*/*Lrrc58* double-mutant mice were yet smaller (12% smaller at E18.5) (Fig. 4C), which accounts for approximately half of the growth defect observed in *Zswim8*^−/−^ mice at this developmental time point. Mice with a maternally inherited *Plagl1* trigger site mutation exhibited normal embryonic growth, consistent with the paternal expression of *Plagl1* mRNA (Supplemental Fig. S11). The growth defect observed in *Plagl1*, *Lrrc58*, and *Plagl1*/*Lrrc58* double-mutant mice persisted through adulthood; at 8 weeks old, mice expressing mutant *Plagl1* or *Lrrc58* were ∼7%–8% smaller, and *Plagl1*/*Lrrc58* double-mutant mice were 14% smaller than control littermates (Fig. 4D–F). These results were consistent with a previously reported growth increase in mice with a deletion of the miRNA cluster containing *Mir-322* and *Mir-503* (Jones et al. 2023) but further demonstrate a causal relationship between TDMD and growth.

## Discussion

In this study, we identified five TDMD trigger sites within four trigger transcripts that direct the degradation of three murine miRNAs. These results, some of which have been reported from parallel efforts in other laboratories (Li et al. 2025; LaVigne et al. 2026), help to triple the number of endogenous TDMD triggers identified in mice. *Plagl1* and *Lrrc58* direct degradation of the related and cotranscribed miR-322 and miR-503, respectively, and *Atp6v1g1* and *Lpar4* collaborate to direct degradation of miR-335-3p. Identifying these trigger transcripts causally linked increased expression of these miRNAs in the *Zswim8* knockout context (i.e., ZSWIM8 sensitivity) to their degradation through the TDMD pathway. All four triggers were active in all examined tissues in the E18.5 embryo, suggesting that they would be active in other contexts with similar or greater expression levels.

While we were preparing our manuscript for publication, others reported identification of the trigger sites in *Plagl1*, *Lrrc58*, and *Atp6v1g1*, as well as the *Plagl1*/*Lrrc58*-dependent growth defect (Li et al. 2025; LaVigne et al. 2026). In addition to independently supporting the results of those parallel studies, we identified and validated in vivo TDMD activity for two trigger sites within *Lpar4* that collaborate with each other and a site in *Atp6v1g1* to direct the degradation of miR-335-3p and, in doing so, help establish the principles that (1) multiple sites on the same transcript can collaborate to direct the degradation of a miRNA, and (2) multiple sites on different transcripts can collaborate to direct the degradation of a miRNA.

The observation that multiple sites can direct the degradation of a miRNA poses challenges for trigger site discovery. In the case of the exceptionally sensitive miRNA miR-335-3p, we could detect partial effects for perturbation of individual TDMD sites. However, in the more typical scenario, in which the miRNA is more modestly ZSWIM8-sensitive, the effect of perturbing a single site would be more difficult to detect, and simultaneous perturbation of multiple candidate sites might be required to reveal their activity. Indeed, even for miR-335-3p, an attempt to validate *Lpar4* as a trigger by disrupting one of its sites was not successful (LaVigne et al. 2026), presumably because of the modest effect of disrupting the single site.

The five trigger sites are within the 3′ UTRs of four protein-coding genes with varied functions. *Atp6v1g1* encodes an essential vacuolar ATPase, *Lpar4* encodes a G protein-coupled receptor, *Plagl1* encodes a zinc finger transcription factor, and *Lrrc58* encodes a recently described E3 ubiquitin ligase substrate adapter involved in cysteine catabolism (Ramage et al. 2025; Xiao et al. 2025). The trigger sites contained within these four transcripts display a wide range of evolutionary conservation. The *Lrrc58* and *Atp6v1g1* trigger sites emerged early in eutherian evolution and have been broadly conserved across placental mammals. *Lpar4* trigger sites also emerged in early eutherians but are somewhat less conserved, though most placental mammals harbor at least one site, and the *Plagl1* trigger sites appear to have emerged only recently in the rodent lineage and perhaps independently within *Afrotheria*.

The substitutions that generate a new trigger site provide a mechanism for an mRNA to acquire two independent functions: the ability to code for a protein and the ability to direct miRNA degradation and thereby indirectly influence the expression of hundreds of transcripts. Mutating trigger sites within either *Plagl1*, *Lrrc58*, *Atp6v1g1*, or *Lpar4* did not significantly change accumulation of the trigger mRNA itself (Supplemental Figs. S4A,B, S6B, S7A,C,D), indicating a resistance to mediating repression of their host mRNA, which was also observed for the miR-7 site in Cyrano, TDMD trigger sites in flies, and the miR-35-42 sites in *tts-2* in *Caenorhabditis elegans* (Kleaveland et al. 2018; Kingston et al. 2022; Grimme et al. 2026). These results indicate that the sites, despite imparting a second, noncoding function to the mRNA, do not also serve double duty to repress both the miRNA and the host mRNA, presumably because the ZSWIM8 CRL acts too rapidly for the AGO–miRNA complex to mediate detectable repression of the trigger.

In principle, our *Plagl1* mutant alleles (both of which disrupted 3′ pairing but not seed-pairing to miR-322) might have acquired sensitivity to miR-322, raising the possibility that miR-322-mediated reduction in PLAGL1 protein contributed to the growth phenotype that we observed. We did not observe a significant decrease in *Plagl1* mRNA levels in the heart, lung, or stomach, but we cannot exclude a subtle reduction in PLAGL1 activity. However, such a contribution was presumably minor because the Mendell laboratory (LaVigne et al. 2026) also observed growth restriction for their *Plagl1* allele, which completely lacked a miR-322 binding site and would therefore not be sensitive to miR-322.

Although previously identified endogenous trigger RNAs are all highly expressed in the contexts in which they mediate TDMD (Bitetti et al. 2018; Kleaveland et al. 2018; Shi et al. 2020; Kingston et al. 2022), our results indicate that this is not a universal property. *Lpar4* causes detectable TDMD at much lower expression levels than previously reported TDMD triggers (Supplemental Fig. S12). Indeed, *Lpar4* appears to degrade miR-335-3p as much as *Atp6v1g1* in the liver, where *Atp6v1g1* is >55-fold more abundant. Further studies will be required to determine whether this unusual potency is due to the particular pairing patterns of the sites in *Lpar4* or whether it is due to other features of this exceptional trigger transcript.

Our computational approach identified all five sites for the three miRNAs of this study within its top-scoring candidates. However, this approach has not been productive for some other ZSWIM8-sensitive miRNAs. This uneven success suggests that not all trigger sites have the features inferred from the initially identified sites used to train our search. Indeed, we observed that *Plagl1*-mediated TDMD is not broadly conserved, whereas *Lpar4*-mediated TDMD does not require high expression levels, and recent reports in nematodes and flies indicate that not all trigger–miRNA pairs possess extensive 3′ complementarity (Donnelly et al. 2022; Hiers et al. 2025). For example, in *C. elegans*, a family of miRNAs is rapidly degraded using a mechanism that does not seem to require extensive pairing to the 3′ region of the miRNA (Donnelly et al. 2022; Grimme et al. 2026). Unbiased genetic or biochemical approaches may reveal triggers for more of the ZSWIM8-sensitive mammalian miRNAs.

ZSWIM8 knockout causes lung and heart defects, perinatal lethality, reduced growth, and upregulation of >40 miRNAs in the developing mouse embryo (Jones et al. 2023; Shi et al. 2023). However, when considering that ZSWIM8-directed ubiquitylation might have additional targets beyond AGO, no causal link between TDMD and these phenotypes had been demonstrated. Our work and parallel work by the Mendell laboratory (LaVigne et al. 2026) causally links the ZSWIM8-dependent growth defect at least partially to target-directed degradation of miR-322 and miR-503. Deletion of the miR-322 and miR-503 trigger sites within *Plagl1* and *Lrrc58* phenocopies 55% of the embryonic growth defect observed in *Zswim8* knockout mice. An ∼23% growth defect was reported previously for a *Plagl1* knockout allele that also resulted in no detectable *Plagl1* mRNA expression (Varrault et al. 2017). These results can now be interpreted in light of the newly found dual roles of the *Plagl1* mRNA. Because loss of *Plagl1* mRNA expression would prevent miRNA decay by the miR-322 TDMD site (Varrault et al. 2017), the magnitude of intrauterine growth restriction reported for *Plagl1* knockout likely results from not only loss of PLAGL1 protein but also loss of miR-322 TDMD. Characterization of other TDMD trigger mutants will shed light on whether other reported phenotypes are also caused by dysregulation of miRNA levels and may reveal other physiological roles for regulated miRNA degradation.

miR-322 and miR-503 are members of the extended miR-16 family, which has many known connections to cell cycling and growth. miR-322 and miR-503 are especially coupled to the cell cycle, as they rapidly decrease in expression when mouse fibroblasts reenter the cell cycle (Rissland et al. 2011). This rapid decrease is an emergent property of the cessation of miR-322 and miR-503 biogenesis upon cell cycle reentry coupled with the inherent instability of these miRNAs (Rissland et al. 2011), which we can now attribute to the *Plagl1* and *Lrrc58* TDMD sites.

Targets of miR-16 family miRNAs include known cell cycle regulators, such as *BCL2*, MAP kinase pathway members, *CYCLIN D1/2/3*, *CYCLIN E1*, *CDC25A*, and *CDK6* (Cimmino et al. 2005; Linsley et al. 2007; Bonci et al. 2008; Liu et al. 2008; Marasa et al. 2009; Rissland et al. 2011). These miRNAs are also deleted or downregulated in various cancer contexts, where cells are rapidly cycling (Calin et al. 2002, 2005; Bandi et al. 2009; Klein et al. 2010; Liu et al. 2019). Conversely, overexpression of miR-16 family members results in accumulation of cells in G1 (Linsley et al. 2007; Liu et al. 2008). Another validated target of the miR-16 family is *insulin-like growth factor 1 receptor* (*IGF1R*) (Llobet-Navas et al. 2014). IGF1 is a regulator of fetal growth and development, and defects in IGF1 and IGF1R impair embryonic growth (Baker et al. 1993; Liu et al. 1993; Powell-Braxton et al. 1993). The upregulation of miR-322 and miR-503 and the consequent enhanced repression of their target mRNAs in TDMD mutant mice presumably mediate growth restriction through the pathways described above, among others.

Our results showing that newly identified triggers, together with their associated miRNAs, fall within imprinted gene networks link TDMD to genomic imprinting and do so in a manner consistent with the parental conflict hypothesis of kinship theory. *Plagl1* mRNA, the trigger for miR-322, is maternally imprinted (Kamiya 2000; Piras et al. 2000), suggesting that it acts in the interests of the paternal chromosomes, which it indeed appears to do by two different means: (1) It encodes a transcription factor that acts in a broader imprinted gene network to enhance intrauterine growth through induction of other imprinted genes, including *Igf2*, *Cdkn1c*, *Gnas*, and *H19* (Varrault et al. 2006, 2017; Iglesias-Platas et al. 2014), and (2) it directs the degradation of miR-322, a growth-inhibiting miRNA (Figs. 2, 4; LaVigne et al. 2026). In another imprinted layer in this regulatory network, *Plagl1* is repressed by miRNAs from the *Mirg* miRNA cluster (Whipple et al. 2020) that are imprinted on the paternal allele (Seitz et al. 2003). An orthogonal connection between TDMD and imprinting is the role of the nonimprinted genes *Atp6v1g1* and *Lpar4* in the degradation of miR-335, a maternally imprinted miRNA expressed within the intron of the maternally imprinted gene *Mest*, which enhances growth in mice (Hiramuki et al. 2015). Many other ZSWIM8-sensitive miRNAs are imprinted (Bartel 2018; Jones et al. 2023; Shi et al. 2023). Determining how more of these presumed regulatory interactions have been leveraged in parental genome conflict will require further investigation. Nonetheless, all evidence seems to indicate that TDMD, like transcriptional and other posttranscriptional regulatory processes (Cleaton et al. 2014; Peters 2014; Tucci et al. 2019), has been a frequent weapon in the conflict between the parental chromosomes.

## Materials and methods

### Computational pipeline for identification of candidate triggers

Computational identification and scoring of candidate TDMD trigger sites was performed through multiple searches. In the first search, sites for each miRNA sequence that are annotated as conserved targets in TargetScanMouse release 8.0 (Agarwal et al. 2015; McGeary et al. 2019) were scored. In a separate search using the same scoring scheme, all canonical seed matches within mRNA 3′ UTRs as well as long noncoding sequences annotated in Ensembl GRCm38 version 102 were scored. Candidate genes were filtered for expression in mouse embryonic fibroblasts (MEFs) (Shi et al. 2020). For each site, a 30 nt region upstream of each site was searched for the pairing configuration with the highest extent of complementarity to the 3′ end of the miRNA. Complementarity was scored with the following scheme to identify sites similar to previously identified TDMD triggers: (1) Each base pair match (after nucleotide 12) was awarded one point; (2) base-pairing to the penultimate or third-to-last nucleotide of the miRNA was awarded an additional 0.5 point; (3) G:U wobble-pairing was not awarded points, except 0.5 point was awarded if a wobble pair was present at the last position of the miRNA; (4) one point was deducted for each gap or mismatch; and (5) offsets >3 were penalized with a 0.5 point penalty per nucleotide >3. In parallel, the base-pairing energy was calculated using the RNAduplex function from the ViennaRNA package for all scored candidate sites (Lorenz et al. 2011).

### Computational analysis of trigger conservation

For each miRNA and TDMD trigger site, the sequence alignment from the mammalian 470 way alignment (https://hgdownload.soe.ucsc.edu/goldenPath/hg38/multiz470way) was downloaded from the UCSC Genome Browser using the Table Browser tool (Karolchik 2004). The mature miRNA sequence for each species was predicted from the genomic sequence. 3′ pairing scores were calculated for each cognate miRNA–trigger site pair using the same computational pipeline as above.

### Tissue culture (cell lines + reagents)

All cells were cultured at 37°C with 5% CO_2_. MEFs and HEK293T cells were cultured in DMEM supplemented with 10% FBS. HFF-1 cells were cultured in DMEM supplemented with 15% FBS. IMR90 cells were cultured in EMEM supplemented with 10% FBS. BJ cells were cultured in EMEM supplemented with 10% FBS. BJ-5ta cells were cultured in a 4:1 mixture of DMEM and medium 199 supplemented with 0.01 mg/mL hygromycin B and 10% FBS. CRISPRi MEF and CRISPRi BJ-5ta cell lines were generated by transducing MEF or BJ-5ta cell lines with a lentivirus encoding a Zim3-dCas9-P2A-GFP fusion protein and sorting for GFP-positive cells (a gift from Jonathan Weissman) (Replogle et al. 2022). All cells were passaged every 3–6 days to maintain confluency between 10% and 75%, except when cells were grown to contact inhibition immediately prior to harvesting.

### Cas9-mediated site deletion

In initial pilot experiments, we observed substantial clone-to-clone variability in miRNA expression levels. To mitigate the potential influence of this variability on assessment of candidate TDMD triggers, we first generated clonal parental cell lines by transfecting MEFs with a PX458-derived plasmid harboring a nontargeting guide and isolating clonal cell lines from the transfected population. These clonal cell lines were used as the parental cell lines for subsequent experiments. Cas9-mediated site deletions were generated by transfecting a clonal parental MEF cell line with two PX458-derived plasmids harboring guides flanking the candidate TDMD site (Supplemental Table S3). *Zswim8* knockout clones were generated by transfecting the same parental clonal MEF cell line with a single PX458-derived plasmid harboring a guide targeting *Zswim8*. Control cell lines were generated by transfecting the same parental clonal MEF cell line with a PX458-derived plasmid harboring a nontargeting guide. For all experiments, single GFP-positive cells were sorted 2 or 3 days after transfection into 96 well plates containing DMEM supplemented with 20% FBS, 50% conditioned media, and penicillin/streptomycin (Gibco). The characterized cell lines were confirmed to be homozygous for the desired mutations by TOPO cloning of genomic DNA amplicons and Sanger sequencing of multiple clones. All clonal cell lines were grown under contact inhibition for at least 5 days before harvesting.

### CRISPRi knockdown

Lentiviral production and transduction were performed as described previously (Shi et al. 2020). Transfer plasmids encoding single guide RNAs were cotransfected with packaging plasmids in HEK293T cells using the reverse transfection technique. For simultaneous knockdown of *Atp6v1g1* and *Lpar4*, the corresponding guides were delivered with a dual-guide transfer plasmid (Replogle et al. 2022).Transfer plasmid (1.4 µg), 0.94 µg of pCMV-dR8.91 packaging plasmid (a gift from Jonathan Weissman), and 0.47 µg of pMD2.G envelope plasmid (Addgene 12259) were transfected per ∼170,000 cells in a 6 well plate using Lipofectamine 2000 and Opti-MEM. After 72 h, the media was collected and centrifuged at 500*g* for 10 min to remove debris. For each well of a 6 well plate, 500 µL of virus-containing media was added to CRISPRi MEFs in culture medium supplemented with polybrene (Santa Cruz Biotechnology) at a final concentration of 1 µg/mL. Plates were centrifuged at 1200*g* for 1.5 h. CRISPRi MEF cells were grown under 4 µg/mL puromycin selection beginning 2 days after transduction. Media was refreshed every 2 days. CRISPRi MEF cells were grown to contact inhibition for at least 5 days before harvesting. CRISPRi knockdown in CRISPRi BJ-5ta cells was performed similarly, except puromycin selection was performed at a final concentration of 1 µg/mL, and cells were not grown under contact inhibition. Knockdown of target genes was confirmed by reverse transcription quantitative PCR (RT-qPCR).

### RNA extraction

Total RNA was extracted from cultured cell lines and mouse tissues using TRI reagent (Thermo Fisher). Cultured cell lines were scraped from culture dishes into TRI reagent. Following euthanasia, mouse tissues were rapidly dissected and flash-frozen in liquid N_2_ in Eppendorf tubes. Frozen tissue was transferred to a 50 mL conical tube, 1–2 mL of TRI reagent was added, and the tissue was homogenized using a TissueRuptor and disposable probes (Qiagen). Following resuspension and/or homogenization, samples were phase-separated with 200 µL of chloroform (J.T. Baker Analytical) for cultured cell lines or 100 µL of 1-bromo-3-chloropropane (Sigma) for tissue. Total RNA was precipitated in isopropanol, washed twice in 75% ethanol, and resuspended in water.

### Small RNA Northern blot

Five micrograms of total RNA was resolved on a denaturing 15% polyacrylamide gel and transferred to Hybond-NX or Hybond-N^+^ membranes (Cytiva) using a semidry transfer apparatus (Bio-Rad). To cross-link RNA to the membrane, the membrane was incubated in a solution of N-(3-dimethylaminopropyl)-N′-ethylcarbodiimide (EDC; Thermo) diluted in 1-methylimidazole for 1 h at 60°C. Radiolabeled DNA or LNA oligonucleotide probes were incubated overnight in ULTRAhyb-oligo hybridization buffer (Invitrogen). Prior to reprobing, hybridized probes were stripped from the membrane by incubation in boiling 0.04% SDS with agitation. A detailed protocol for small RNA Northern blot analysis is available at http://bartellab.wi.mit.edu/protocols.html. Results were analyzed on a Typhoon phosphorimager (Cytiva) and quantified using ImageQuant TL (v8.1.0.0). Northern blot probe sequences and hybridization temperatures are listed in Supplemental Table S3.

### RT-qPCR

For RT-qPCR experiments, cDNA was prepared from 0.5–1 µg of total RNA using the QuantiTect reverse transcript kit (Qiagen) according to the manufacturer's instructions. qPCR experiments were performed on a Roche LightCycler II instrument using SYBR Green I qPCR master mix. qPCR primer sequences are listed in Supplemental Table S3.

### mRNA sequencing and analysis

For *Plagl1/Lrrc58* mutant and wild-type heart samples, RNA-seq libraries were prepared from total RNA using the Watchmaker RNA library preparation kit (Watchmaker Genomics). Ribosomal RNA depletion was performed using RiboDepletion oligos (Qiagen) by mixing 14 µL of RNA input, 1 µL of FastSelect reagent (Qiagen), and 10 µL of Frag & Prime buffer (Watchmaker Genomics) and incubation under the following conditions: 10 min at 85°C, 2 min at 75°C, 2 min at 70°C, 2 min at 65°C, 2 min at 60°C, 2 min at 55°C, 2 min at 37°C, 2 min at 25°C, and held at 4°C. Samples were prepared from the first strand synthesis step of the Watchmaker RNA library preparation kit (Watchmaker Genomics) onward, according to the manufacturer's instructions. Libraries were multiplexed using xGen UDI primers (IDT) and sequenced on the Illumina NovaSeq platform with paired-end reads. Gene expression quantification was performed using salmon with the ‐‐gcBias and ‐‐validateMappings options to map to the mouse transcriptome (GRCm38 version 102) (Patro et al. 2017). Only reads mapping to mRNAs or lncRNAs were considered for depth normalization. Differential gene expression analysis was performed using DESeq2 v1.38.3 without the use of the lfcShrink function (Love et al. 2014). For Plagl1 stomach, Lrrc58 lung, and Atp6v1g1 and Lpar4 heart and lung mutant and wild-type samples, RNA-seq libraries were prepared from purified RNA and sequenced by Plasmidsaurus. Gene counts were analyzed with DESeq2 v1.38.3 without the use of the lfcShrink function.

### sRNA-seq

Small RNA sequencing libraries were prepared from 5 µg of total RNA. miR-427-5p (0.5 fmol; *Xenopus tropicalis*) and 0.5 fmol of lsy-6-3p (*C. elegans*) were added to each sample as spike-ins. Small RNA species were isolated by excising the gel fragment migrating between 18 and 32 nt radiolabeled internal standards on a 15% polyacrylamide urea gel. Size-selected RNA was eluted from the gel, precipitated in ethanol, and ligated to a preadenylated 3′ adapter (AppNNNNCTGTCTCTTATACACATCTCCGAGCddC) using T4 RNA ligase 2 KQ mutant (NEB) in a reaction supplemented with 10% polyethylene glycol (PEG 8000; NEB). The 3′ adapter had four random sequence positions at its 5′ end to reduce ligation bias. Ligated small RNAs were isolated on a 10% polyacrylamide urea gel, precipitated in ethanol, and ligated to a 5′ adapter (CCUACACGACGCUCUUCCGAUCUNNNN) using T4 RNA ligase I (NEB) in a reaction supplemented with 10% PEG. The 5′ adapter had four random sequence positions at its 3′ end to reduce ligation bias. Ligated small RNAs were isolated on an 8% polyacrylamide urea gel, precipitated in ethanol, and reverse-transcribed with SuperScript III (Invitrogen). The resulting cDNA was amplified using KAPA HiFi DNA polymerase (Kapa Biosystems). Amplified DNA was purified on a 90% formamide and 8% acrylamide gel and submitted for sequencing on the Illumina HiSeq or NovaSeq platform. A step-by-step protocol for constructing libraries for small RNA sequencing is available at http://bartellab.wi.mit.edu/protocols.html.

Adapter sequences were trimmed from reads using Cutadapt (Martin 2011). Reads were filtered for quality using fastq_quality_filter (FastX toolkit; http://hannonlab.cshl.edu/fastx_toolkit/index.html) with the parameters “–q 30 –p 100.”

To assign processed sequencing reads to miRNAs, the first 19 nt of each read was matched to a dictionary of miRNA sequences downloaded from TargetScan release 8.0 (Agarwal et al. 2015; McGeary et al. 2019), requiring no mismatches between the read and the miRNA dictionary. Reads mapping to the spike-in miRNAs and markers were removed for further analysis. Differential expression analysis was performed using DESeq2 v1.38.3 without the use of the lfcShrink function (Love et al. 2014).

### miRNA targeting

miRNA targeting analysis was performed as described by Stefano et al. (2026). Briefly, miRNA target predictions were downloaded from TargetScan release 8.0 (Agarwal et al. 2015; McGeary et al. 2019), and repression of predicted miRNA targets was analyzed in differential expression data. Genes expressed at <10 TPMs across samples were excluded. Two sets of predicted targets were analyzed: all predicted targets and top predicted targets (10% of targets with the lowest cumulative weighted context++ scores) (Agarwal et al. 2015). Each set of targets was compared with a control group of genes not predicted to be targets of the miRNA family under consideration. The nontarget cohort was selected by sampling transcripts at a 1:1 ratio with targets, matching the distribution of 3′ UTR lengths between the target and nontarget cohorts. The distribution of log_2_ fold changes (DESeq2 output) in samples relative to *Plagl1* WT, *Lrrc58* het samples was compared with that of the nontarget cohort. Statistical significance was assessed using a Mann–Whitney *U*-test. The degree of repression is represented by subtracting the median log_2_ fold change of the target set from that of its corresponding nontarget set. The mean degree of repression and median *P*-value across 21 iterations of the above analysis are reported. Supplemental Figure S5C displays a representative cumulative distribution function (derived from the iteration that generated the median *P*-value for the all targets set), with only the nontarget set corresponding to the “all targets” set shown for simplicity.

### Mouse husbandry

Mice were housed at the Whitehead Institute for Biomedical Research in accordance with protocols approved by the Massachusetts Institute of Technology Committee on Animal Care. Mice were housed in a 12 h light/dark cycle (light from 07:00 to 19:00) with free access to food and water. Euthanasia of adults was performed by CO_2_ inhalation, and euthanasia of embryos was performed by rapid decapitation over ice.

### Generation of mutant mice

Mutant mice were generated by the Whitehead Institute Genetically Engineered Models Core. Mice with mutations in *Atp6v1g1, Lpar4, Plagl1*, and *Lrrc58* 3′ UTRs were generated by injecting or electroporating C57BL/6J embryos with Cas9 protein complexed with a sgRNA designed to cut within the regions of the 3′ UTR predicted to engage in TDMD (see Supplemental Table S3). For *Plagl1* and *Lrrc58*, 1 cell embryos were electroporated with Cas9, sgRNAs, and HDR donor oligos. For *Lpar4*, Cas9, sgRNAs, and HDR donor oligos were injected into 1 cell embryos. For *Atp6v1g1*, Cas9 and sgRNAs were injected into one blastomere of 2 cell embryos. F_0_ mice containing the resulting deletions and mutations were backcrossed with C57BL/6J mice for at least two generations to obtain the desired heterozygous mice that were then used to generate embryos and adult mice used in this study. Mutant lines were maintained by breeding to C57BL/6J or heterozygotes. No substantial phenotypic differences were observed between mice bearing different mutant alleles in *Plagl1* or *Lrrc58*, so mutant alleles were used interchangeably in this study. Mutant mice generated in this study have been submitted to The Jackson Laboratory (*Atp6v1g1*^*−50*^ [041300], *Lpar4*^*−197*^ [041301], *Lpar4*^*−173*^ [041302], *Lpar4*^*−69+3*6^ [041303], *Plagl1*^*−22+7*^ [041171], *Plagl1*^*−8*^ [041172], *Lrrc58*^*−402*^ [041174], *Lrrc58*^*−134*^ [041175], and *Lrrc58*^*TDMD_mut1*^ [041173]).

### Genotyping

Initial genotyping of mutant mice generated for this study was performed by extracting genomic DNA using the HotSHOT method (Truett et al. 2000), amplifying regions of interest by PCR using the primers listed in Supplemental Table S3, and analyzing genomic sequences by nanopore sequencing. *Atp6v1g1* mutant mice were genotyped by gel electrophoresis of PCR amplicons containing the TDMD site for all subsequent studies. For other mutant mice, automated genotyping was performed by Transnetyx.

### Timed mating and tissue collection

Pregnant females were euthanized by CO_2_ inhalation at embryonic day 18.5 (E18.5; 18 days after inspection of a vaginal plug). Embryos were rapidly dissected over ice and weighed after gently blotting dry. Embryos were decapitated and tissues were dissected in ice-cold PBS. Tissues were flash-frozen and carried forward for RNA extraction as described.

### Mouse weight analysis

Mutant mice and their littermates were weighed at E18.5 or 8 weeks of age. Statistical testing for mouse weights was performed separately for each sex by using a linear mixed-effects model, with the litter as a random effect and genotype as a linear effect. Reported *P*-values are the pairwise comparisons between genotypes with Tukey correction.

### Data availability

All raw and processed sequencing data will be deposited at the NCBI Gene Expression Omnibus under accession number GSE318680.

## Supplemental Material

Supplement 1

Supplement 2

Supplement 3

Supplement 4
