# Supplementary material for: mRNA 3′ UTRs direct microRNA degradation to participate in imprinted gene networks and regulate growth
Source: Genes Dev. 2026 Apr 1;40(7-8):586–603. doi: 10.1101/gad.353479.125 (PMC13041716; doi:10.1101/gad.353479.125)
Supplement: Supplement 1 [file Supplemental_Material.pdf]

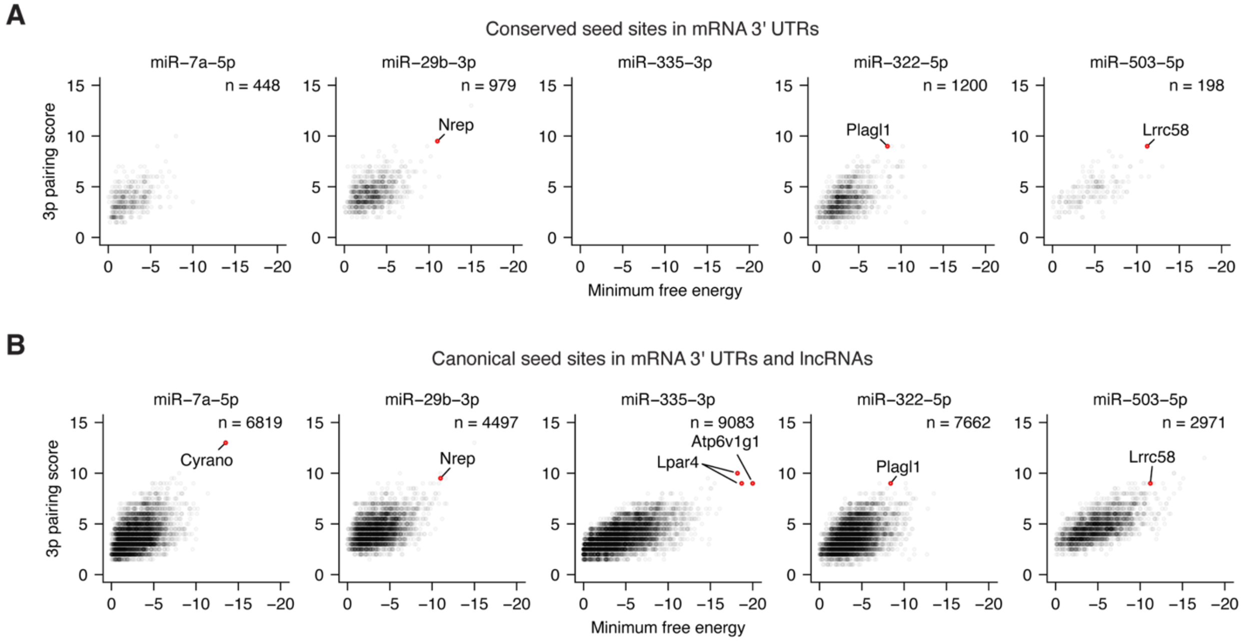

Supplemental Figure S1. A computational pipeline predicts TDMD trigger sites for miR-335-3p, miR-322-5p, and miR-503-5p.

A) Results of a version of the trigger-site prediction pipeline that scores conserved sites. For each conserved seed site (8mer, 7mer-A1, 7mer-m8) in 3′ UTRs, the 3′-pairing score is plotted as a function of predicted 3′-pairing energy. Validated and newly identified TDMD trigger sites are labeled and highlighted in red. miR-335-3p was not analyzed using this pipeline because it had no conserved sites annotated by TargetScan, as it was considered a passenger strand when conserved sites were annotated by TargetScan, and TargetScan does not predict conserved sites of passenger strands.

B) Results of a version of the trigger-site prediction pipeline that also scores all non-conserved seed sites in 3′ UTRs and all seed sites in lncRNAs; otherwise, as in A.

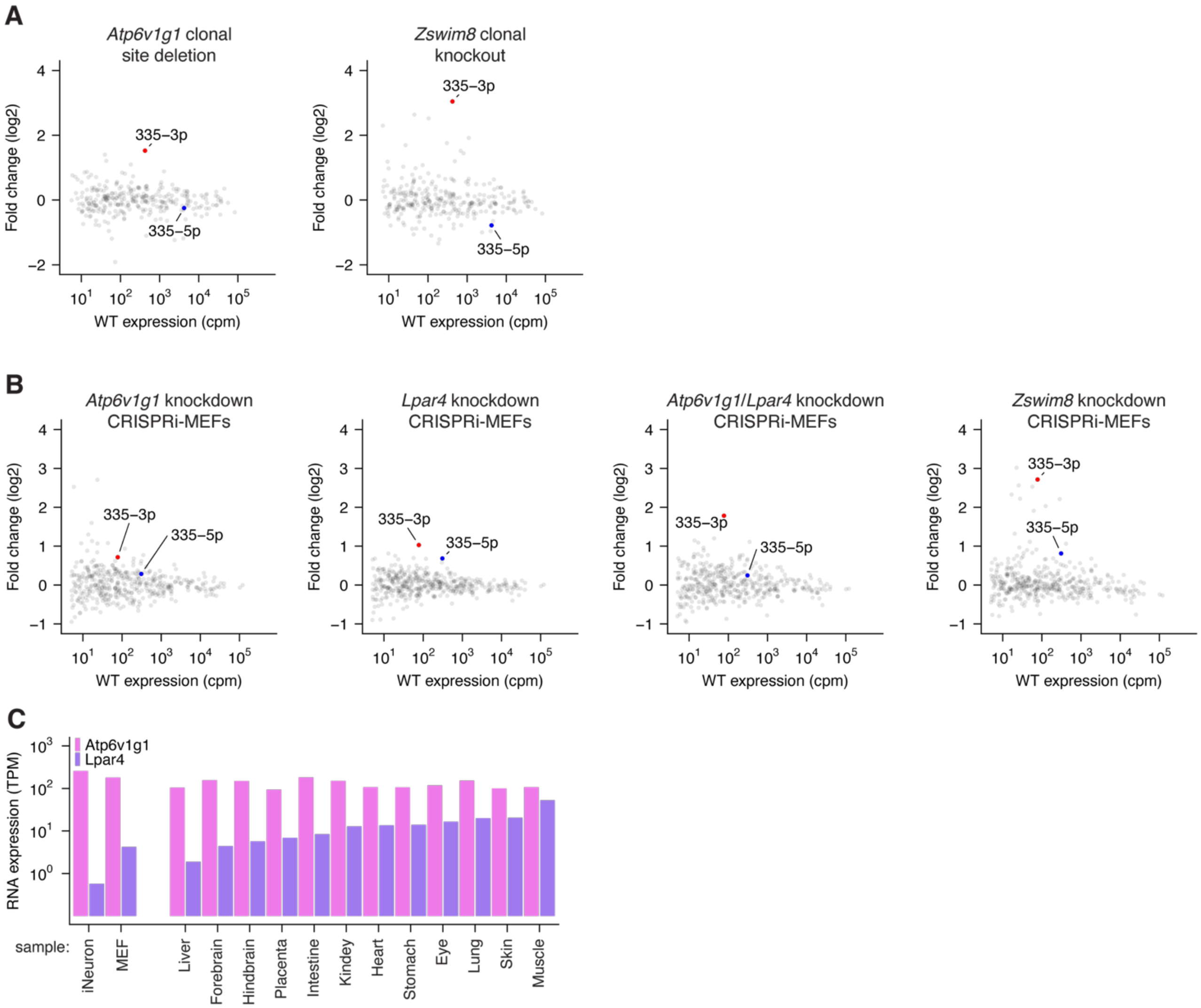
A
Atp6v1g1 clonal site deletion
Zswim8 clonal knockout
335–3p
335–5p
Fold change (log2)
WT expression (cpm)
B
Atp6v1g1 knockdown CRISPRi-MEFs
Lpar4 knockdown CRISPRi-MEFs
Atp6v1g1/Lpar4 knockdown CRISPRi-MEFs
Zswim8 knockdown CRISPRi-MEFs
C
Atp6v1g1
Lpar4
RNA expression (TPM)
sample:
iNeuron
MEF
Liver
Forebrain
Hindbrain
Placenta
Intestine
Kindey
Heart
Stomach
Eye
Lung
Skin
Muscle

Supplemental Figure S2. sRNA-seq from cell lines validates *Atp6v1g1* and *Lpar4* as endogenous TDMD triggers.

A) sRNA-seq results showing the changes in miRNA levels observed upon disruption of either the miR-335-3p trigger site within *Atp6v1g1* or the *Zswim8* gene. Results from 3–4 clonal lines per genotype were analyzed using DESeq2. The point for miR-335-3p is in red, and the point for the coproduced miR-335-5p strand is in blue.

B) sRNA-seq results showing changes in miRNA levels observed when CRISPRi-MEFs were transduced with guides targeting either *Atp6v1g1*, *Lpar4*, both *Atp6v1g1* and *Lpar4*, or *Zswim8*, compared to cells targeted with non-targeting guides. Results from two biological replicates were analyzed using DESeq2; otherwise, as in (A).

C) Relative expression of *Atp6v1g1* and *Lpar4* mRNAs, as quantified by RNA-seq in mouse cell lines and embryonic tissues (Shi et al. 2023).

A

Atp6v1g1 tctcctttcttaca**ggtcaggggcgttatctatgaaaaa**gtagaagtagctattagtaactcctatgaagaagcagccagcctt

–50 nt tctcctttcttaca**ggt**--aagaagcagccagcctt

B

Lpar4 acagtatttgtgccc**aggtcaggagtaaattgaaaaa**gtaagtgaatagaatagtagcagcaagatatcttaga...

–173 nt acagtatttgtgccc---

–69 nt acagtatttgtgccc**aggtcaggagtaaattgaaaaa**gtaagtgaatagaatagtagcagcaagatatcttaga

–197 nt acagtatttgtgccc---

Lpar4 ...gcttatattagtagtttttaaggtggtggttagatagctgtaattttgaaatccatactctcttctgtacattt...

–173 nt --

–69 nt gcttatattagtagtttttaaggtggtggttagatagctgtaattttgaaatccatactctcttctgtaca---

–197 nt --

Lpar4 ...tggagcacattgtagccaaggcgctgctgaatttgtgctc**aggtcgggagcatattgaaaaa**gatgtgtacata

–173 nt --**aggtcgggagcatattgaaaaa**gatgtgtacata

–69 nt ---------------------------------AATTCAGCAGCGGTTGGCTACAATGTGCTCCAAtgtacata

–197 nt ---atgtgtacata

C

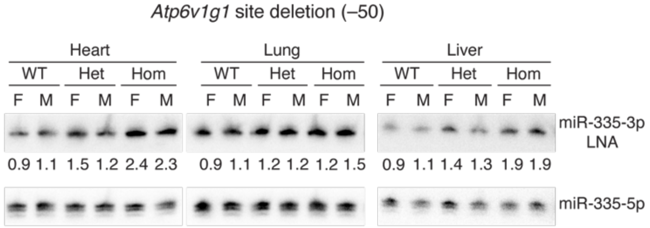

D

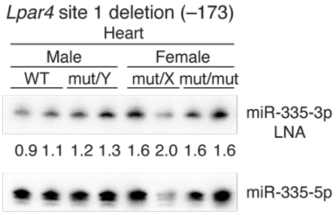

E

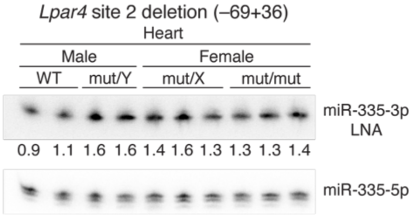

F

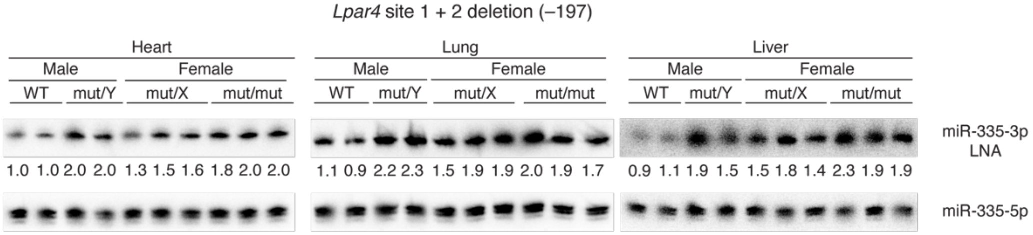

Supplemental Figure S3. Validation of *Atp6v1g1* and *Lpar4* as TDMD triggers for miR-335 in mice.

A) Wild-type and mutant sequences of mouse *Atp6v1g1*. For each gene, the wild-type sequence is shown above the mutant sequence. TDMD sites are in bold; gaps are indicated by dashes; substitutions are in red, and predicted Cas9 cleavage sites are indicated by scissors above the wild type sequences.

B) Wild-type and mutant sequences for *Lpar4*; otherwise, as in A.

C) Validation of *Atp6v1g1* as a TDMD trigger for miR-355-3p in mice. Shown is a northern blot resolving total RNA of heart, lung, and liver of E18.5 mice, probed for miR-335-3p. Mice were either wild-type (WT), heterozygous, or homozygous for a deletion of the TDMD trigger site in *Atp6v1g1*. Numbers below the lanes indicate the relative fold change, which was obtained by normalizing first to the value of the miR-335-5p loading control and then to the average of the WT samples.

D–E) Validation of *Lpar4* as a TDMD trigger for miR-355-3p in mice. Shown are northern blots of RNA from E18.5 heart of *Lpar4* mutant mice in which either TDMD site 1 ($Lpar4^{-173}$) or TDMD site 2 ($Lpar4^{-69+36}$) has been deleted (panels D and E, respectively); otherwise, as in panel C.

F) Effect of deleting both trigger sites within *Lpar4*. Shown are northern blots of RNA from E18.5 heart, lung, and liver for *Lpar4* mutant mice in which both TDMD sites have been deleted ($Lpar4^{-197}$). Otherwise, as in panel C.

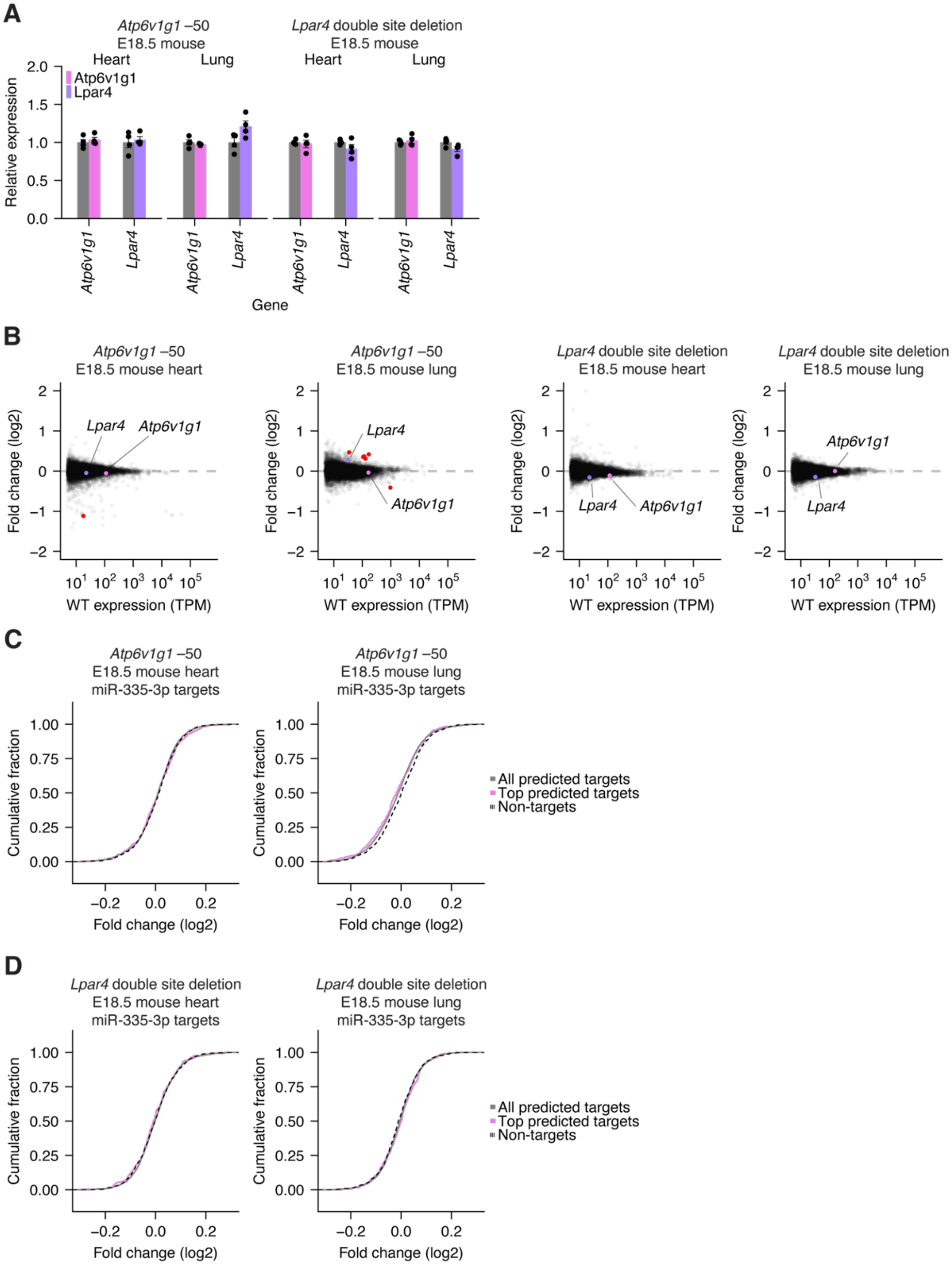
A
Atp6v1g1 –50
E18.5 mouse
Lpar4 double site deletion
E18.5 mouse
Heart
Lung
Heart
Lung
Atp6v1g1
Lpar4
Relative expression
2.0
1.5
1.0
0.5
0.0
Atp6v1g1
Lpar4
Atp6v1g1
Lpar4
Atp6v1g1
Lpar4
Atp6v1g1
Lpar4
Gene
B
Atp6v1g1 –50
E18.5 mouse heart
Atp6v1g1 –50
E18.5 mouse lung
Lpar4 double site deletion
E18.5 mouse heart
Lpar4 double site deletion
E18.5 mouse lung
Fold change (log2)
Lpar4
Atp6v1g1
WT expression (TPM)
C
Atp6v1g1 –50
E18.5 mouse heart
miR-335-3p targets
Atp6v1g1 –50
E18.5 mouse lung
miR-335-3p targets
Cumulative fraction
1.00
0.75
0.50
0.25
0.00
−0.2
0.0
0.2
Fold change (log2)
All predicted targets
Top predicted targets
Non-targets
D
Lpar4 double site deletion
E18.5 mouse heart
miR-335-3p targets
Lpar4 double site deletion
E18.5 mouse lung
miR-335-3p targets
Cumulative fraction
Fold change (log2)
All predicted targets
Top predicted targets
Non-targets

Supplemental Figure S4. The expression of *Atp6v1g1*, *Lpar4*, and miR-335-3p targets in mutant and wild-type tissues.

A) The influence of triggers sites within *Atp6v1g1* or *Lpar4* on host transcript expression. Plotted are the relative expression levels of *Atp6v1g1* or *Lpar4* in the mutant mouse tissue compared to wild-type littermate control tissue. No significant changes in expression were detected, as determined with DESeq2.

B) RNA-seq results showing changes in mRNA levels observed in mutants of the TDMD trigger site mutant mice for the *Atp6v1g1* or *Lpar4* sites compared to their littermates. For each mutant mouse strain, results from 8 animals (4 per genotype) were analyzed using DESeq2.

C–D) The influence of trigger sites within *Atp6v1g1* or *Lpar4* (panels C or D, respectively) on levels of miR-335-3p predicted targets in vivo. Plotted are cumulative distributions of mRNA fold changes observed in the mutant E18.5 mouse heart or lung relative to wild-type for all predicted targets and top 10% of predicted targets, as determined by TargetScan (Agarwal et al. 2015), and their corresponding control cohorts, matched for 3′ UTR length. For simplicity, only the control cohort corresponding to all predicted targets is displayed (dashed line). The selection of control cohorts was repeated 21 times, and the cohort with the median *P* value (Mann–Whitney U test) is shown here, with this *P* value and the distributions of differences in the median fold changes reported in Fig. 2J.

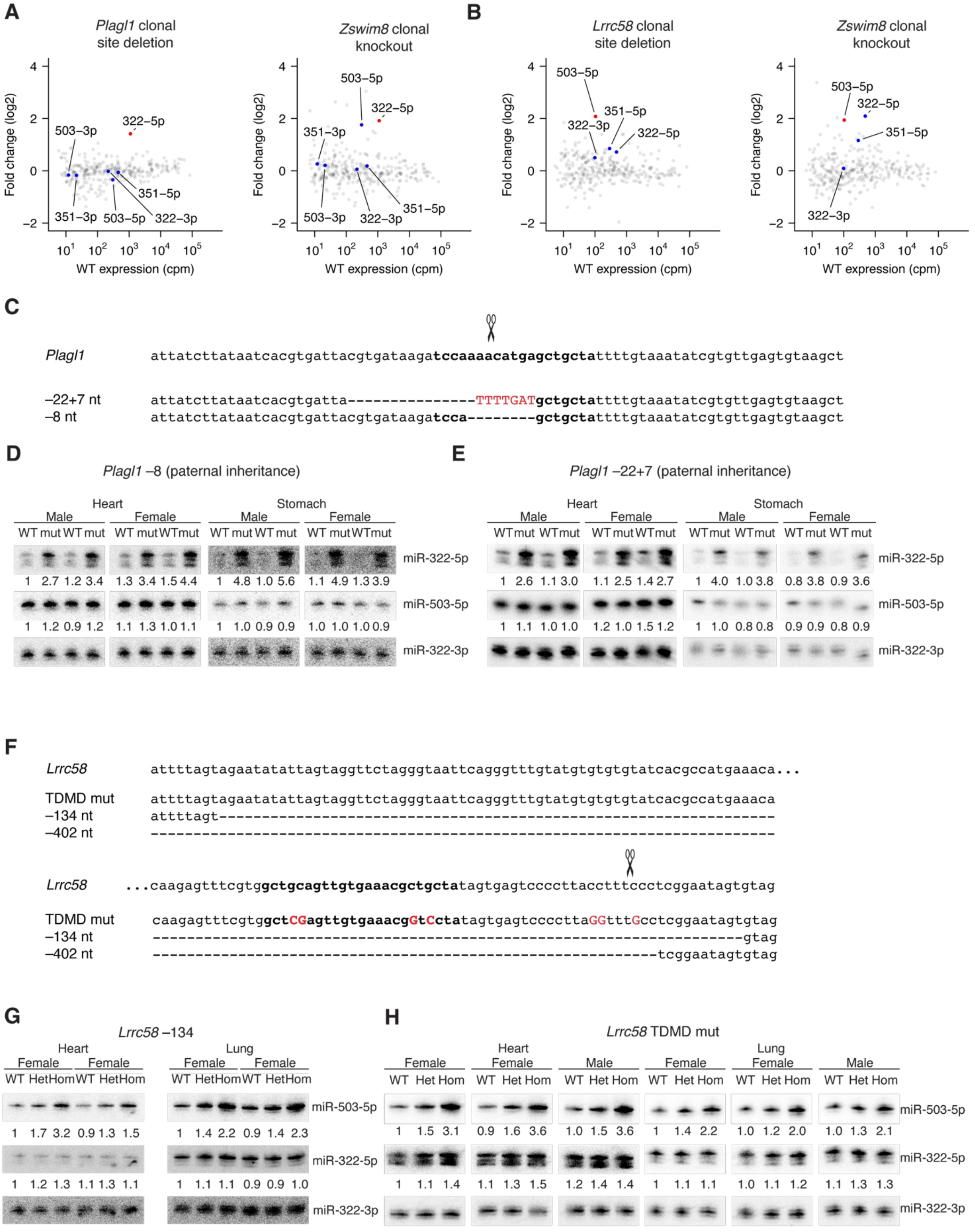
A
Plagl1 clonal site deletion
Zswim8 clonal knockout
B
Lrrc58 clonal site deletion
Zswim8 clonal knockout
Fold change (log2)
WT expression (cpm)
503–5p
503–3p
322–5p
322–3p
351–5p
351–3p
C
Plagl1
attatcttataatcacgtgattacgtgataagatccaaaacatgagctgctatttttgtaaatatcgtgttgagtgtaagct
–22+7 nt
attatcttataatcacgtgatta---------------TTTTGATgctgctatttttgtaaatatcgtgttgagtgtaagct
–8 nt
attatcttataatcacgtgattacgtgataagatcca--------gctgctatttttgtaaatatcgtgttgagtgtaagct
D
Plagl1 –8 (paternal inheritance)
E
Plagl1 –22+7 (paternal inheritance)
Heart
Stomach
Male
Female
WT mut WT mut
miR-322-5p
miR-503-5p
miR-322-3p
F
Lrrc58
attttagtagaatatattagtaggttctagggtaattcagggtttgtatgtgtgtgtatcacgccatgaaaca...
TDMD mut
attttagtagaatatattagtaggttctagggtaattcagggtttgtatgtgtgtgtatcacgccatgaaaca
–134 nt
attttagt--
–402 nt
Lrrc58
...caagagtttcgtggctgcagttgtgaaacgctgctatagtgagtccccttacctttccctcggaatagtgtag
TDMD mut
caagagtttcgtggctCGagttgtgaaacgGtCctatagtgagtccccttaGGtttGcctcggaatagtgtag
–134 nt
---gtag
–402 nt
--tcggaatagtgtag
G
Lrrc58 –134
H
Lrrc58 TDMD mut
Heart
Lung
Female
Male
WT Het Hom
miR-503-5p
miR-322-5p
miR-322-3p

Supplemental Figure S5. Validation of *Plagl1* and *Lrrc58* as TDMD triggers for miR-322 and miR-503, respectively.

A) sRNA-seq results showing the changes in miRNA levels observed upon disruption of either the *Zswim8* gene or the miR-322 TDMD trigger site within *Plagl1*. The point for miR-322-5p is in red, and points for coproduced miR-322-3p, miR-503-5p, miR-503-3p, miR-351-5p, and miR-351-3p are in blue. Results from 3–4 clonal lines per genotype were analyzed using DESeq2.

B) sRNA-seq results showing the changes in miRNA levels observed upon disruption of either the *Zswim8* gene or the miR-503 TDMD trigger site in *Lrrc58*. The point for miR-503-5p is in red, and points for coproduced miR-322-5p, miR-322-3p, miR-503-3p, miR-351-5p, and miR-351-3p are in blue. Results from 2–3 clonal lines per genotype were analyzed using DESeq2.

C) Wild-type and mutant sequences of mouse *Plagl1*; otherwise, as in Supplemental Fig. S3A.

D–E) Validation of *Plagl1* as a TDMD trigger for miR-322-5p in mice. Shown are northern blots of RNA from heart and lung of E18.5 mice with different alleles of trigger-site mutants in *Plagl1* (panels D and E, respectively). Analyses of sex- and litter-matched samples are shown for each of the indicated genotypes. Numbers below the lanes indicate the relative fold change, which was obtained by normalizing first to the value of the loading control (miR-322-3p) and then to the average of the WT samples.

F) Wild-type and mutant sequences of mouse *Lrrc58*; otherwise, as in Supplemental Fig. S3A.

G–H) Validation of *Lrrc58* as a TDMD trigger for miR-503-5p in mice. Shown are northern blots of RNA from heart and lung of E18.5 mice with different alleles of trigger-site mutants in *Lrrc58* (panels G and H, respectively). Otherwise, as in (D–E).

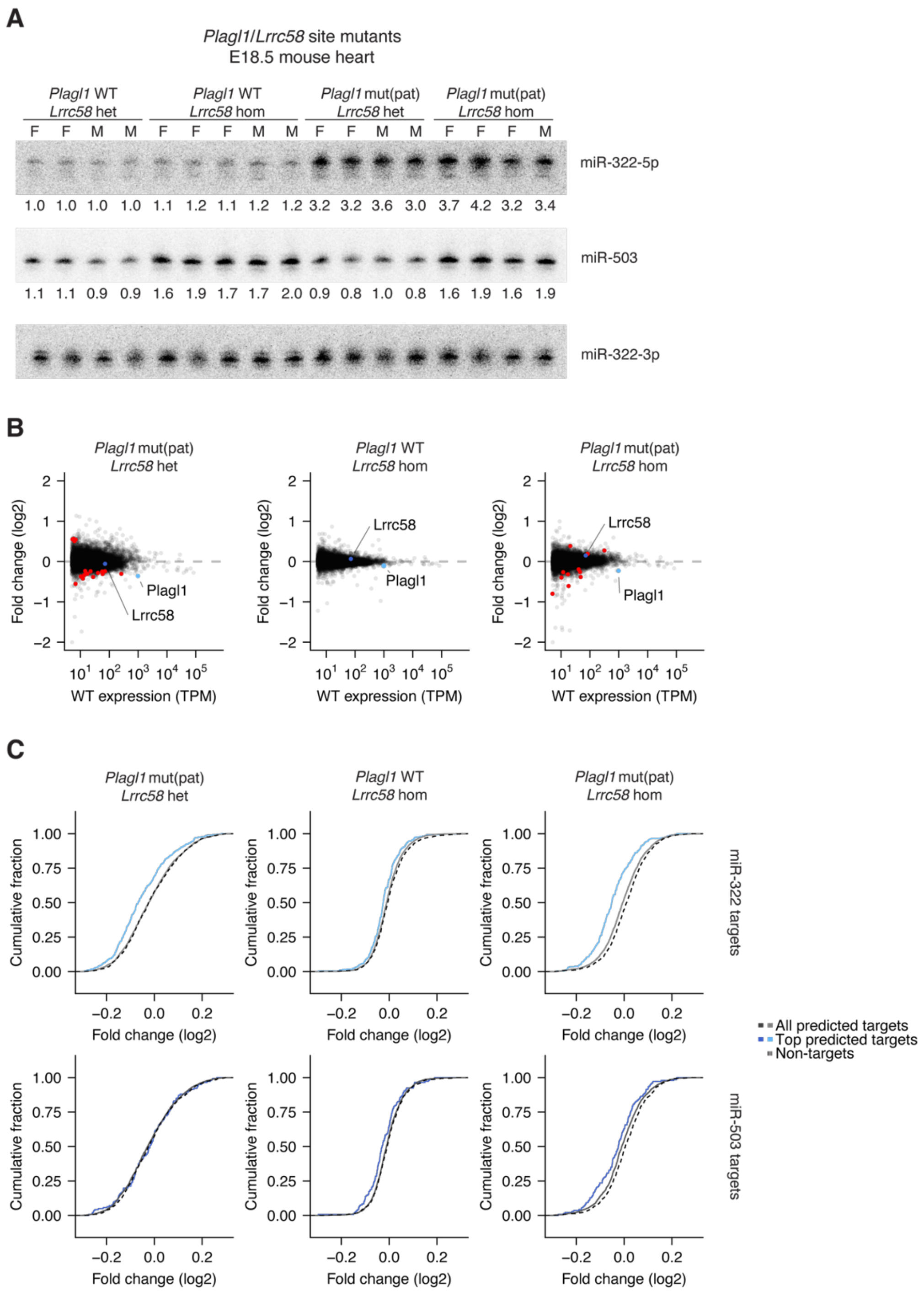
A
Plagl1/Lrrc58 site mutants
E18.5 mouse heart
Plagl1 WT
Lrrc58 het
Plagl1 WT
Lrrc58 hom
Plagl1 mut(pat)
Lrrc58 het
Plagl1 mut(pat)
Lrrc58 hom
F F M M F F F M M F F M M F F F M
miR-322-5p
1.0 1.0 1.0 1.0 1.1 1.2 1.1 1.2 1.2 3.2 3.2 3.6 3.0 3.7 4.2 3.2 3.4
miR-503
1.1 1.1 0.9 0.9 1.6 1.9 1.7 1.7 2.0 0.9 0.8 1.0 0.8 1.6 1.9 1.6 1.9
miR-322-3p
B
Fold change (log2)
WT expression (TPM)
Plagl1
Lrrc58
C
Cumulative fraction
Fold change (log2)
miR-322 targets
miR-503 targets
All predicted targets
Top predicted targets
Non-targets

Supplemental Figure S6. *Plagl1* and *Lrrc58* TDMD trigger sites influence miRNA and mRNA expression in mice.

A) Effects of mutating *Plagl1* and *Lrrc58* trigger sites in mice. Shown are northern blots of RNA from heart of E18.5 mice with the indicated trigger site mutants in *Plagl1* and *Lrrc58*. Genotype and sex are indicated above the blots (WT, wild-type; het, heterozygous mutant; hom, homozygous mutant; mut(pat), heterozygous with paternally inherited mutant allele; F, female; M, male).

B) RNA-seq results showing changes in mRNA levels observed in mutants of the TDMD trigger sites in *Plagl1* (*Plagl1* mut(pat), *Lrrc58* het), *Lrrc58* (*Plagl1* WT, *Lrrc58* hom), and both *Plagl1* and *Lrrc58* (*Plagl1* mut(pat) *Lrrc58* hom), compared to *Plagl1* WT, *Lrrc58* het littermates. Results from 24 animals (5–7 per genotype) were analyzed using DESeq2.

C) The influence of trigger sites within *Plagl1* and *Lrrc58* on levels of miR-503-5p and miR-322-5p predicted targets in vivo. Plotted are cumulative distributions of mRNA fold changes observed in the mutant E18.5 mouse heart relative to wild-type for all predicted targets and top 10% of predicted targets, as determined by TargetScan (Agarwal et al. 2015), and their corresponding control cohorts, matched for 3′ UTR length. For simplicity, only the control cohort corresponding to all predicted targets is displayed (dashed line). The selection of control cohorts was repeated 21 times, and the cohort with the median *P* value (Mann–Whitney U test) is shown here, with this *P* value and the distributions of differences in the median
fold changes reported in Fig. 2I.

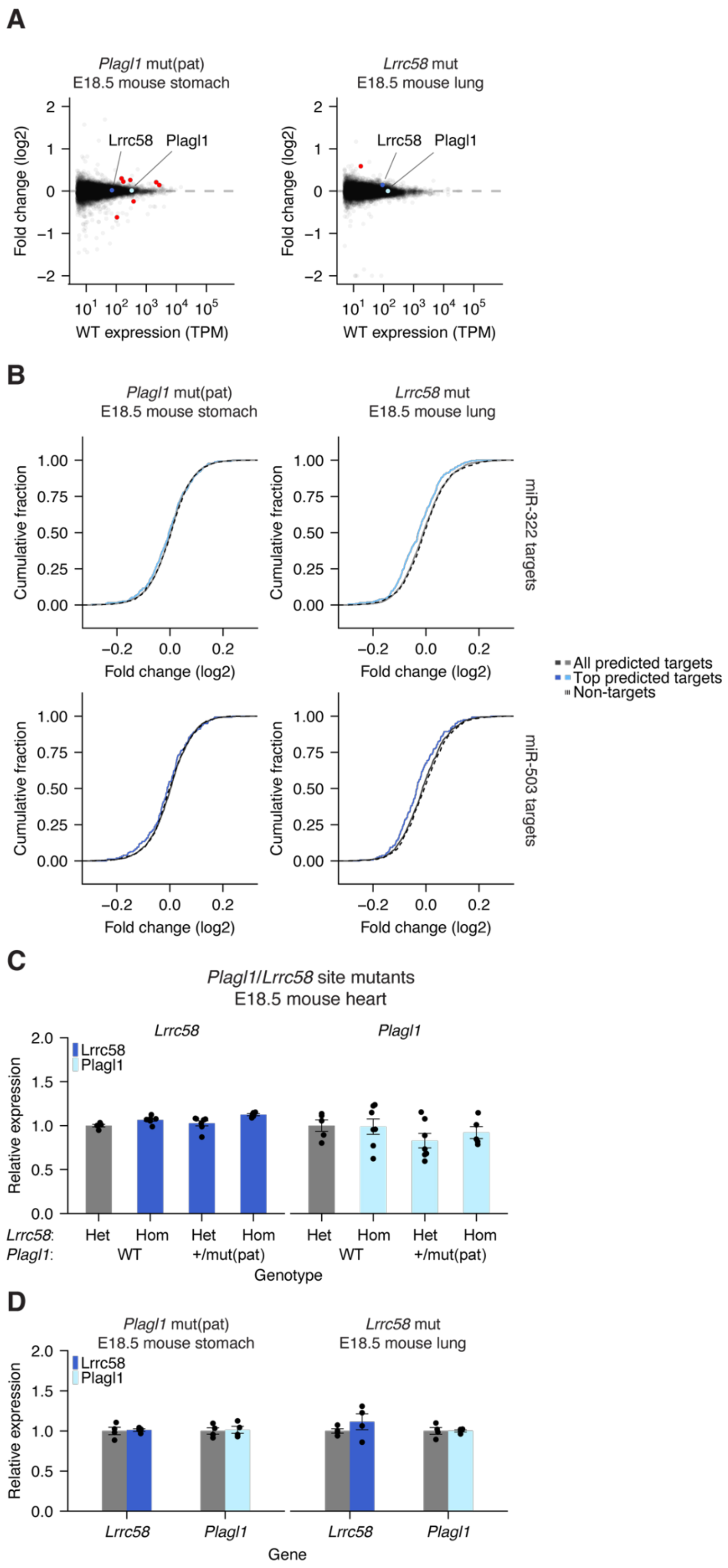
A
Plagl1 mut(pat)
E18.5 mouse stomach
Lrrc58 mut
E18.5 mouse lung
Lrrc58
Plagl1
Fold change (log2)
WT expression (TPM)
B
Cumulative fraction
Fold change (log2)
miR-322 targets
miR-503 targets
All predicted targets
Top predicted targets
Non-targets
C
Plagl1/Lrrc58 site mutants
E18.5 mouse heart
Lrrc58
Plagl1
Relative expression
Lrrc58:
Plagl1:
Het
Hom
WT
+/mut(pat)
Genotype
D
Plagl1 mut(pat)
E18.5 mouse stomach
Lrrc58 mut
E18.5 mouse lung
Relative expression
Gene

Supplemental Figure S7. The expression of *Plagl1*, *Lrrc58*, and miR-322 and miR-503 targets in mutant and wild-type tissues.

A) RNA-seq results showing changes in mRNA levels observed in trigger site mutant mice for the *Plagl1* or *Lrrc58* sites compared to their littermates. For each mutant mouse strain, results from 8 animals (4 per genotype) were analyzed using DESeq2.

B) The influence of trigger sites within *Plagl1* or *Lrrc58* (left or right, respectively) on levels of miR-503-5p or miR-322-5p predicted targets in vivo. Plotted are cumulative distributions of mRNA fold changes observed in the mutant E18.5 mouse lung or stomach relative to wild-type for all predicted targets and top 10% of predicted targets, as determined by TargetScan (Agarwal et al. 2015), and their corresponding control cohorts, matched for 3′ UTR length. For simplicity, only the control cohort corresponding to all predicted targets is displayed (dashed line). The selection of control cohorts was repeated 21 times, and the cohort with the median *P* value (Mann–Whitney U test) is shown here, with this *P* value and the distributions of differences in the median fold changes reported in Fig. 2J.

C) The influence of triggers sites within *Plagl1* or *Lrrc58* on host transcript expression. Plotted are the relative expression levels of *Plagl1* or *Lrrc58* in mutants of the TDMD trigger sites in *Plagl1* (*Plagl1* mut(pat), *Lrrc58* het), *Lrrc58* (*Plagl1* WT, *Lrrc58* hom), and both *Plagl1* and *Lrrc58* (*Plagl1* mut(pat) *Lrrc58* hom), compared to *Plagl1* WT, *Lrrc58* het littermates. Results from 24 animals (5–7 per genotype) were analyzed using DESeq2. No significant changes in expression were detected, as determined with DESeq2.

D) The influence of triggers sites within *Plagl1* or *Lrrc58* on host transcript expression. Same as (C), but the relative expression levels of *Plagl1* or *Lrrc58* in mutant E18.5 mouse lung or stomach.

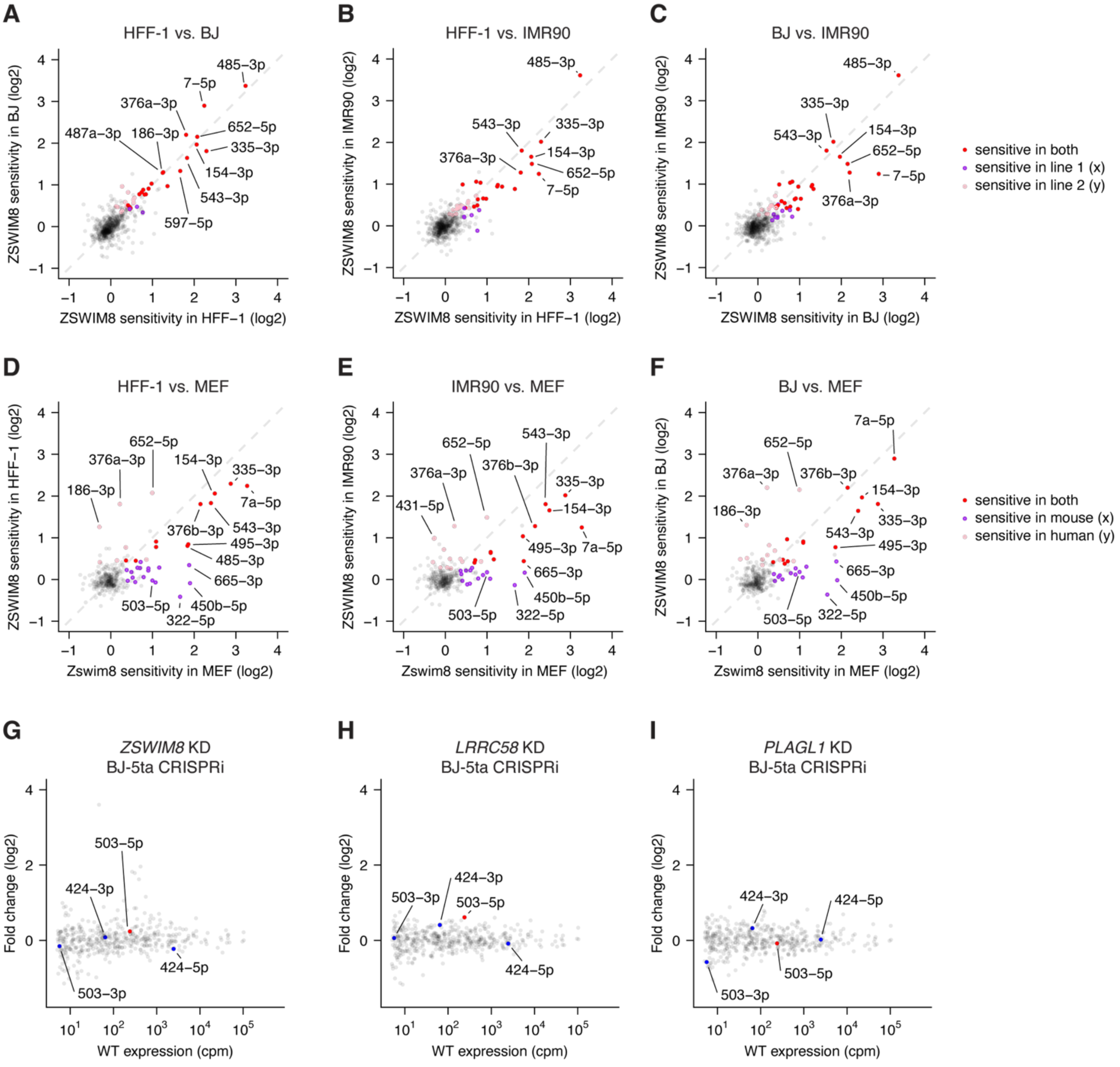

A
HFF-1 vs. BJ
ZSWIM8 sensitivity in BJ (log2)
ZSWIM8 sensitivity in HFF−1 (log2)
485−3p
7−5p
376a−3p
487a−3p
186−3p
652−5p
335−3p
154−3p
543−3p
597−5p
B
HFF-1 vs. IMR90
ZSWIM8 sensitivity in IMR90 (log2)
ZSWIM8 sensitivity in HFF−1 (log2)
485−3p
543−3p
335−3p
154−3p
376a−3p
652−5p
7−5p
C
BJ vs. IMR90
ZSWIM8 sensitivity in IMR90 (log2)
ZSWIM8 sensitivity in BJ (log2)
485−3p
335−3p
543−3p
154−3p
652−5p
7−5p
376a−3p
sensitive in both
sensitive in line 1 (x)
sensitive in line 2 (y)
D
HFF-1 vs. MEF
ZSWIM8 sensitivity in HFF−1 (log2)
Zswim8 sensitivity in MEF (log2)
652−5p
376a−3p
154−3p
335−3p
186−3p
7a−5p
376b−3p
543−3p
495−3p
485−3p
665−3p
503−5p
450b−5p
322−5p
E
IMR90 vs. MEF
ZSWIM8 sensitivity in IMR90 (log2)
Zswim8 sensitivity in MEF (log2)
543−3p
652−5p
376b−3p
376a−3p
335−3p
431−5p
154−3p
495−3p
7a−5p
665−3p
450b−5p
503−5p
322−5p
F
BJ vs. MEF
ZSWIM8 sensitivity in BJ (log2)
Zswim8 sensitivity in MEF (log2)
7a−5p
652−5p
376a−3p
376b−3p
154−3p
186−3p
335−3p
543−3p
495−3p
665−3p
450b−5p
503−5p
322−5p
sensitive in both
sensitive in mouse (x)
sensitive in human (y)
G
ZSWIM8 KD
BJ-5ta CRISPRi
Fold change (log2)
WT expression (cpm)
503−5p
424−3p
424−5p
503−3p
H
LRRC58 KD
BJ-5ta CRISPRi
Fold change (log2)
WT expression (cpm)
424−3p
503−3p
503−5p
424−5p
I
PLAGL1 KD
BJ-5ta CRISPRi
Fold change (log2)
WT expression (cpm)
424−3p
424−5p
503−5p
503−3p

Supplemental Figure S8. ZSWIM8-sensitve miRNAs are abundant in human fibroblasts.

A–C) Abundant ZSWIM8-sensitive miRNAs in three human fibroblast lines. Shown are pairwise comparisons of miRNA fold-changes observed in three different human fibroblast cell lines upon ZSWIM8 knockout, as calculated by DESeq2 (Table S1). Points for miRNAs with statistically significant increases in both cell lines are red (Wang and Bartel 2023). Points for miRNAs with statistically significant increases in only one cell line are purple or pink.

D–F) Comparison of ZSWIM8 sensitivities observed between human and mouse. For each miRNA conserved from human to mouse, the fold-change observed in human fibroblast lines upon ZSWIM8 knockout is plotted as a function of the change reported in mouse embryonic fibroblasts (Shi et al. 2023). Colors are as in A. G–H) Conservation of TDMD trigger activity to human cells. Shown are sRNA-seq results plotting miRNA fold changes observed in BJ-5ta CRISPRi human fibroblasts upon knockdown (KD) of either *ZSWIM8* (panel G), *LRRC58* (panel H), or *PLAGL1* (panel I). Results from two biological replicates were analyzed by DESeq2; otherwise, as in Supplemental Fig. S2B.

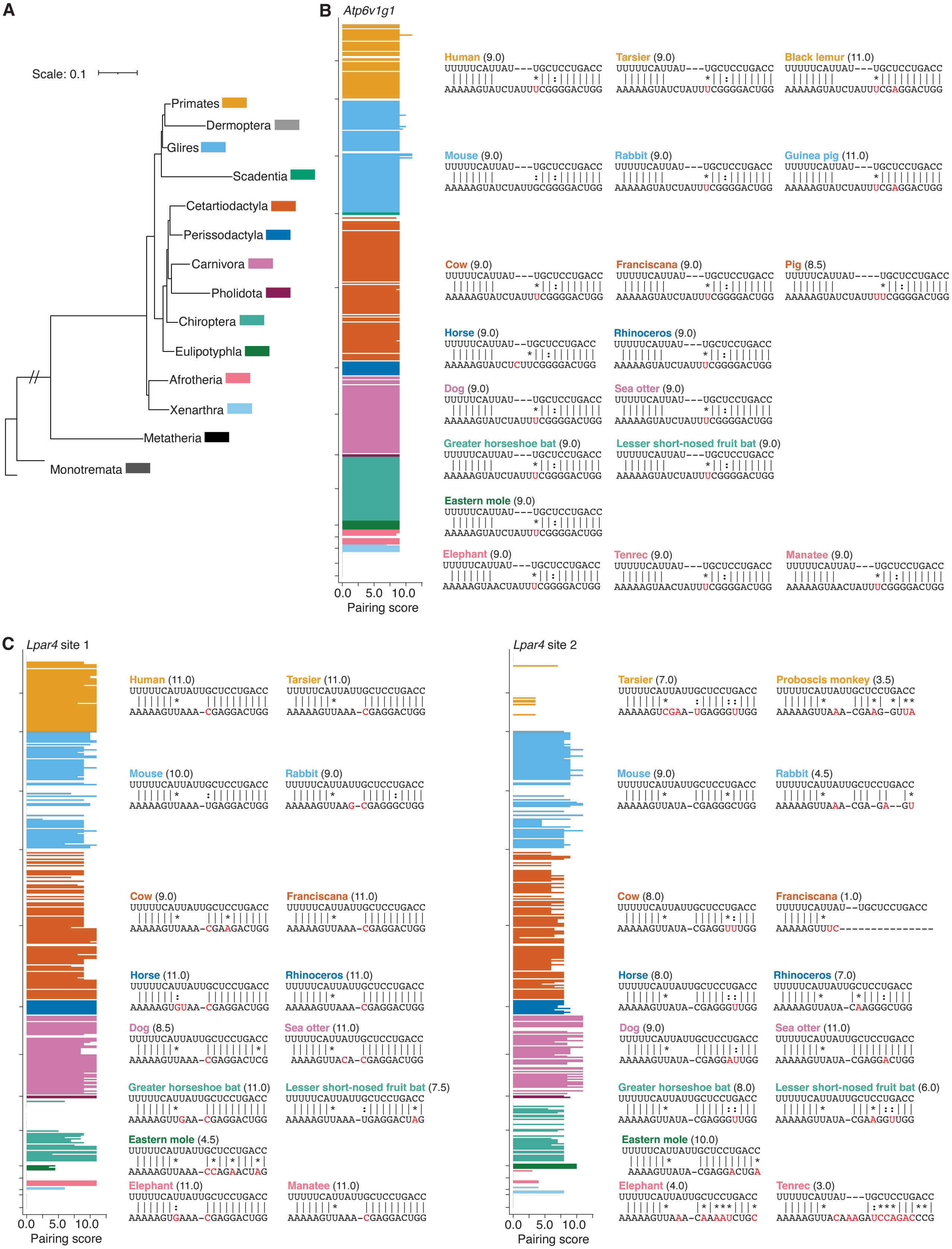
A
Scale: 0.1
Primates
Dermoptera
Glires
Scadentia
Cetartiodactyla
Perissodactyla
Carnivora
Pholidota
Chiroptera
Eulipotyphla
Afrotheria
Xenarthra
Metatheria
Monotremata
B
Atp6v1g1
Pairing score
C
Lpar4 site 1
Lpar4 site 2
Pairing score

Supplemental Figure S9. TDMD sites in *Atp6v1g1* and *Lpar4* are evolutionarily conserved.

A) Simplified phylogenetic tree of mammalian lineages, based on a 470-species whole-genome alignment (https://hgdownload.soe.ucsc.edu/goldenPath/hg38/multiz470way/) (Letunic and Bork 2024).

B) Evolutionary conservation of extensive 3′ complementarity within the miR-335-3p TDMD trigger site in *Atp6v1g1*, across a 470-species mammalian alignment. Each row in the plot corresponds to the 3′ pairing score of our computational pipeline for a species in the 470-way alignment, colored according to clade, as indicated in (A). Missing rows indicate species lacking an orthologous seed match to miR-335-3p. Representative pairing diagrams are shown on the right, with positions that differ from the mouse sequences colored in red. Vertical lines indicate W–C–F pairing; a colon indicates G:U wobble pairing; an asterisk indicates a mismatch.

C) As in (B) but for the 2 miR-335-3p trigger sites within *Lpar4*.

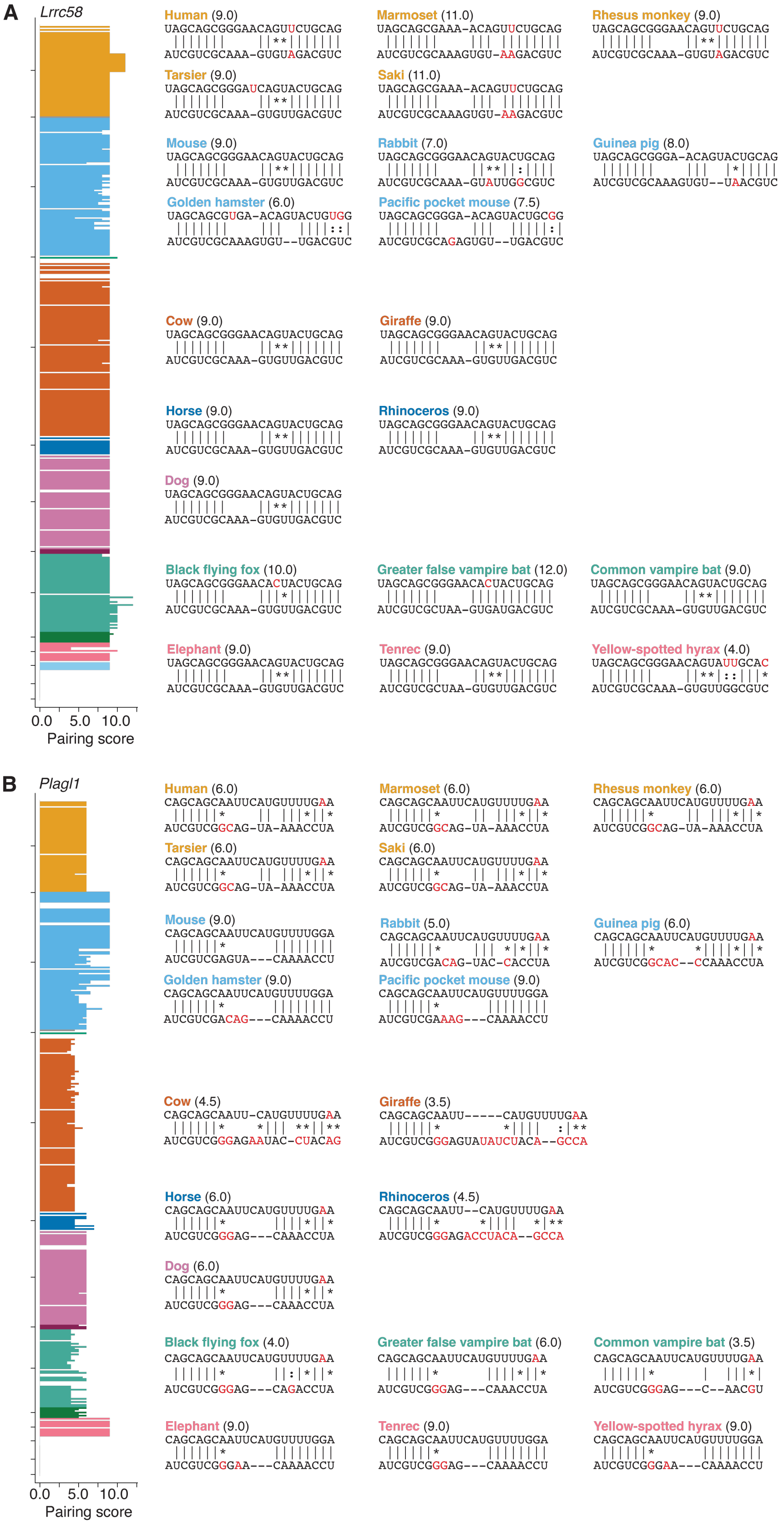
A
Lrrc58
Human (9.0)
UAGCAGCGGGAACAGUUCUGCAG
AUCGUCGCAAA-GUGUAGACGUC
Marmoset (11.0)
UAGCAGCGAAA-ACAGUUCUGCAG
AUCGUCGCAAAGUGU-AAGACGUC
Rhesus monkey (9.0)
UAGCAGCGGGAACAGUUCUGCAG
AUCGUCGCAAA-GUGUAGACGUC
Tarsier (9.0)
UAGCAGCGGGAUCAGUACUGCAG
AUCGUCGCAAA-GUGUUGACGUC
Saki (11.0)
UAGCAGCGAAA-ACAGUUCUGCAG
AUCGUCGCAAAGUGU-AAGACGUC
Mouse (9.0)
UAGCAGCGGGAACAGUACUGCAG
AUCGUCGCAAA-GUGUUGACGUC
Rabbit (7.0)
UAGCAGCGGGAACAGUACUGCAG
AUCGUCGCAAA-GUAUUGGCGUC
Guinea pig (8.0)
UAGCAGCGGGA-ACAGUACUGCAG
AUCGUCGCAAAGUGU--UAACGUC
Golden hamster (6.0)
UAGCAGCGUGA-ACAGUACUGUGG
AUCGUCGCAAAGUGU--UGACGUC
Pacific pocket mouse (7.5)
UAGCAGCGGGA-ACAGUACUGCGG
AUCGUCGCAGAGUGU--UGACGUC
Cow (9.0)
UAGCAGCGGGAACAGUACUGCAG
AUCGUCGCAAA-GUGUUGACGUC
Giraffe (9.0)
UAGCAGCGGGAACAGUACUGCAG
AUCGUCGCAAA-GUGUUGACGUC
Horse (9.0)
UAGCAGCGGGAACAGUACUGCAG
AUCGUCGCAAA-GUGUUGACGUC
Rhinoceros (9.0)
UAGCAGCGGGAACAGUACUGCAG
AUCGUCGCAAA-GUGUUGACGUC
Dog (9.0)
UAGCAGCGGGAACAGUACUGCAG
AUCGUCGCAAA-GUGUUGACGUC
Black flying fox (10.0)
UAGCAGCGGGAACACUACUGCAG
AUCGUCGCAAA-GUGUUGACGUC
Greater false vampire bat (12.0)
UAGCAGCGGGAACACUACUGCAG
AUCGUCGCUAA-GUGAUGACGUC
Common vampire bat (9.0)
UAGCAGCGGGAACAGUACUGCAG
AUCGUCGCAAA-GUGUUGACGUC
Elephant (9.0)
UAGCAGCGGGAACAGUACUGCAG
AUCGUCGCAAA-GUGUUGACGUC
Tenrec (9.0)
UAGCAGCGGGAACAGUACUGCAG
AUCGUCGCUAA-GUGUUGACGUC
Yellow-spotted hyrax (4.0)
UAGCAGCGGGAACAGUAUUGCAC
AUCGUCGCAAA-GUGUUGGCGUC
0.0 5.0 10.0
Pairing score
B
Plagl1
Human (6.0)
CAGCAGCAAUUCAUGUUUUGAA
AUCGUCGGCAG-UA-AAACCUA
Marmoset (6.0)
CAGCAGCAAUUCAUGUUUUGAA
AUCGUCGGCAG-UA-AAACCUA
Rhesus monkey (6.0)
CAGCAGCAAUUCAUGUUUUGAA
AUCGUCGGCAG-UA-AAACCUA
Tarsier (6.0)
CAGCAGCAAUUCAUGUUUUGAA
AUCGUCGGCAG-UA-AAACCUA
Saki (6.0)
CAGCAGCAAUUCAUGUUUUGAA
AUCGUCGGCAG-UA-AAACCUA
Mouse (9.0)
CAGCAGCAAUUCAUGUUUUGGA
AUCGUCGAGUA---CAAAACCU
Rabbit (5.0)
CAGCAGCAAUUCAUGUUUUGAA
AUCGUCGACAG-UAC-CACCUA
Guinea pig (6.0)
CAGCAGCAAUUCAUGUUUUGAA
AUCGUCGGCAC--CCAAACCUA
Golden hamster (9.0)
CAGCAGCAAUUCAUGUUUUGGA
AUCGUCGACAG---CAAAACCU
Pacific pocket mouse (9.0)
CAGCAGCAAUUCAUGUUUUGGA
AUCGUCGAAAG---CAAAACCU
Cow (4.5)
CAGCAGCAAUU-CAUGUUUUGAA
AUCGUCGGGAGAAUAC-CUACAG
Giraffe (3.5)
CAGCAGCAAUU-----CAUGUUUUGAA
AUCGUCGGGAGUAUAUCUACA--GCCA
Horse (6.0)
CAGCAGCAAUUCAUGUUUUGAA
AUCGUCGGGAG---CAAACCUA
Rhinoceros (4.5)
CAGCAGCAAUU--CAUGUUUUGAA
AUCGUCGGGAGACCUACA--GCCA
Dog (6.0)
CAGCAGCAAUUCAUGUUUUGAA
AUCGUCGGGAG---CAAACCUA
Black flying fox (4.0)
CAGCAGCAAUUCAUGUUUUGAA
AUCGUCGGGAG---CAGACCUA
Greater false vampire bat (6.0)
CAGCAGCAAUUCAUGUUUUGAA
AUCGUCGGGAG---CAAACCUA
Common vampire bat (3.5)
CAGCAGCAAUUCAUGUUUUGAA
AUCGUCGGGAG---C--AACGU
Elephant (9.0)
CAGCAGCAAUUCAUGUUUUGGA
AUCGUCGGGAA---CAAAACCU
Tenrec (9.0)
CAGCAGCAAUUCAUGUUUUGGA
AUCGUCGGGAG---CAAAACCU
Yellow-spotted hyrax (9.0)
CAGCAGCAAUUCAUGUUUUGGA
AUCGUCGGGAA---CAAAACCU
0.0 5.0 10.0
Pairing score

Supplemental Figure S10. The TDMD sites in *Lrrc58* is more conserved than that in *Plagl1*.

A) Evolutionary conservation of extensive 3′ complementarity within the miR-503 TDMD trigger site in *Lrrc58,* across a 470-species mammalian alignment (https://hgdownload.soe.ucsc.edu/goldenPath/hg38/multiz470way/). Missing rows indicate species lacking an orthologous seed match to miR-503. Otherwise, as in Supplemental Fig. S7A.

B) As in (A) but for the miR-322/424 site within *Plagl1*.

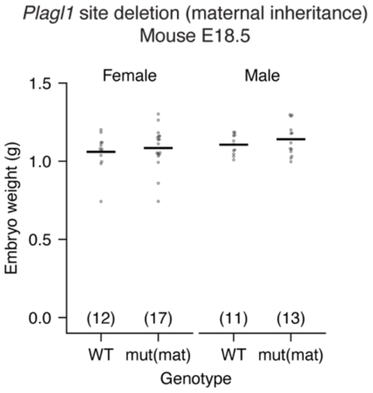

Supplemental Figure S11. Maternal inheritance of *Plagl1* trigger site mutations does not affect embryo growth.

Shown are weights of E18.5 mouse embryos with either a wild-type (WT) or a mutant miR-322 trigger site in the maternal *Plagl1* allele. “mut(mat)” indicates a maternally inherited *Plagl1* allele with a mutation in the miR-322 trigger site. The number of animals for each genotype is indicated in parentheses. Statistical testing was performed using a mixed-linear-effects model. No statistically significant difference was observed.

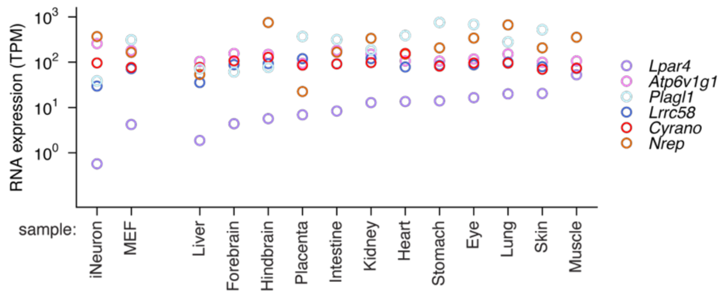

Supplemental Figure S12. Expression of mammalian TDMD triggers can vary.

Relative expression of the six known endogenous mammalian TDMD trigger transcripts, as quantified by RNA-seq in mouse cell lines and embryonic tissues (Shi et al. 2023).
